# Synthesis and Evaluation of ^68^Ga-Labeled NT(6–13) Analogs Incorporating Non-Canonical Amino Acid Substitutions at Tyr^11^ for Targeting NTSR1 in Various Solid Malignancies

**DOI:** 10.1021/acsomega.6c02356

**Published:** 2026-04-29

**Authors:** Simranjeet Kaur, Stefan Mair, Wing Sum Lau, Jutta Zeisler, François Bénard, Kuo-Shyan Lin, Joseph Lau

**Affiliations:** † Department of Basic and Translational Research, 8144 BC Cancer Research Institute, Vancouver, BC V5Z1L3, Canada; ‡ Department of Radiology, University of British Columbia, Vancouver, BC V5Z1M9, Canada

## Abstract

Targeting neurotensin receptor 1 (NTSR1), which is overexpressed in several solid malignancies, remains a promising strategy for molecular imaging. Neurotensin (NT), the endogenous ligand for NTSR1, binds to NTSR1 with subnanomolar affinity but suffers from rapid degradation due to proteolytic cleavage at the Arg^8^–Arg^9^, Pro^10^–Tyr^11^, and Tyr^11^–Ile^12^ peptide bonds. To address this limitation, we designed a series of NT(6–13) analogs by introducing noncanonical amino acids at the Tyr^11^ position to improve receptor binding and metabolic stability. The N-terminus of the strongest binder was further modified by N-terminal acetylation to optimize pharmacokinetics. The synthesized compounds were compared against [^68^Ga]­Ga-NT-20.3, a clinically investigated agent. Based on an *in vitro* competition radioligand-binding assay using PC-3 cells, Ga-SK01001 and Ga-SK01014, among the tested NT(6–13) analogs, exhibit binding affinity values in the low-nanomolar range (*K*_i_ < 2 nM). ^68^Ga-labeling was conducted in HEPES (2 M, pH 5.0) buffer, and the radiolabeled products were obtained in 22–60% decay-corrected radiochemical yields with >190 GBq/μmol molar activity and >95% radiochemical purity. [^68^Ga]­Ga-SK01014 demonstrated significantly higher internalization and reduced efflux compared to [^68^Ga]­Ga-NT-20.3 (reference tracer). In vivo PET imaging in PC-3 tumor-bearing NRG mice revealed that [^68^Ga]­Ga-SK01014 had similar tumor uptake compared to [^68^Ga]­Ga-NT-20.3 (10.0 ± 2.48 vs 9.43 ± 0.73%ID/g at 1 h postinjection), while demonstrating a marked reduction in renal accumulation (2.88 ± 0.64 vs 9.65 ± 1.15%ID/g, *p* < 0.01). Receptor specificity was validated by blocking studies, which resulted in a 92.7% reduction in tumor uptake. Notably, despite exhibiting lower plasma stability at 15 min postinjection (34.0 ± 5.5% intact for [^68^Ga]­Ga-SK01014 vs 64.1 ± 5.9% for [^68^Ga]­Ga-NT-20.3), [^68^Ga]­Ga-SK01014 maintained efficient tumor targeting. This suggests that, in addition to metabolic stability, both early receptor engagement and rapid internalization also play a critical role in determining tumor uptake. Overall, this study identifies [^68^Ga]­Ga-SK01014 as a promising NTSR1-targeted imaging agent with improved renal clearance and provides insight into the balance between stability and receptor-driven tumor uptake, guiding the future design of neurotensin-based theranostic agents.

## Graphical abstract

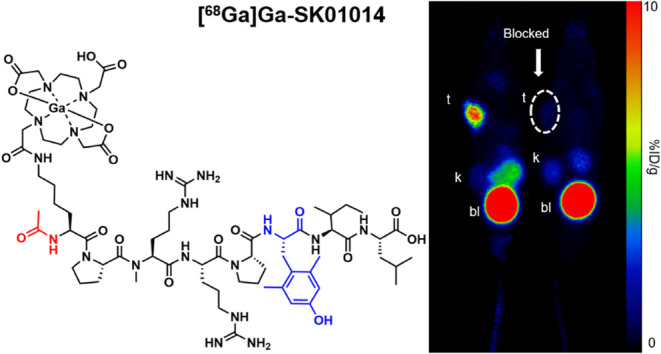

## 1 Introduction

Neurotensin (NT) is a 13-amino-acid peptide that acts as a neuromodulator in the central nervous system, where it induces potent analgesic and hypothermic effects, and as an endocrine modulator in the gastrointestinal tract, affecting motility as well as pancreatic and biliary secretions.^1^ These effects are mediated through neurotensin receptors NTSR1 (high-affinity) and NTSR2 (low-affinity), which belong to the G-protein-coupled receptor (GPCR) superfamily.^2^ At the cellular level, NT signaling through NTSR1 can drive pro-oncogenic processes. NT–NTSR1 activation has been implicated in enhanced cell proliferation and DNA synthesis, as well as increased cell migration and angiogenesis. NTSR1 has emerged as an attractive target for drug development due to its overexpression in a variety of cancers,^3–8^ including pancreatic ductal adenocarcinoma (PDAC),^9,10^ breast cancer,^11–13^ prostate cancer,^14,15^ lung cancer,^16^ colorectal carcinoma,^17,18^ and gastrointestinal stromal tumors.^19,20^ High NTSR1 expression in tumors is associated with less favorable outcomes and poor survival.

From a radiopharmaceutical development perspective, most NTSR1-targeting agents are derived from truncated NT analogs.^21–24^ The major limitation of this approach is the metabolic lability of the endogenous ligand. NT(8–13) (Arg^8^-Arg^9^-Pro^10^-Tyr^11^-Ile^12^-Leu^13^), the minimum peptide sequence needed for target engagement and bioactivity, has a biological half-life of less than 2 min due to cleavage at Arg^8^-Arg^9^, Pro^10^-Tyr^11^, and Tyr^11^-Ile^12^ peptide bonds.^25,26^ Therefore, stabilization of these cleavage sites is crucial to ensure sufficient payload delivery. The best-characterized peptide-based agent is [^68^Ga]­Ga-NT-20.3, an NT(6–13) analog, which is stabilized through N-terminal acetylation, and the incorporation of NMeArg and Tle substitutions at positions 8 and 12, respectively^27,28^ (Figure 1). In preclinical models, [^68^Ga]­Ga-NT-20.3 was able to differentiate pancreatic ductal adenocarcinoma (PDAC) from pancreatitis, supporting its potential as a positron emission tomography (PET) imaging agent.^27,28^ A first-in-human imaging study was conducted in three PDAC patients; however, sensitivity and image contrast (tumor/nontumor uptake) were poor,^29^ suggesting room for tracer optimization.

**Figure 1:**
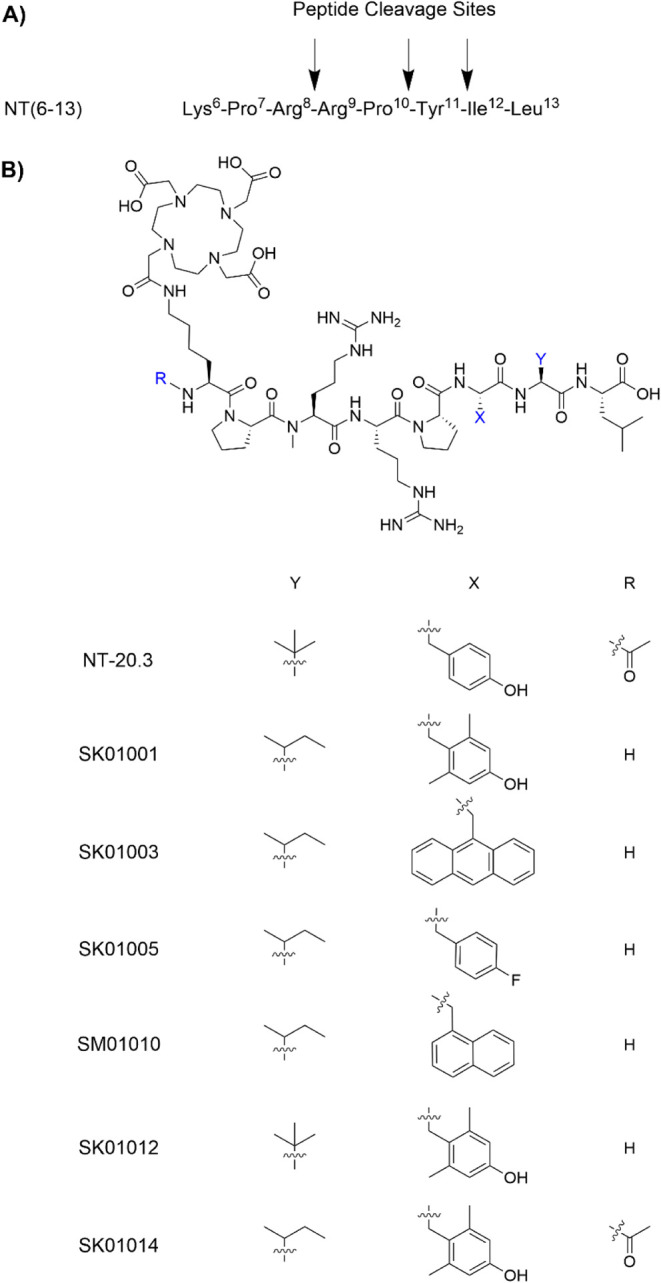
(A) The potential cleavage sites are shown by black arrows in the NT(6–13) peptide sequence. (B) The chemical structures of NT-20.3, SK01001, SK01003, SK01005, SM01010, SK01012, and SK01014 with substitutions at Ile^12^ (Y), Tyr^11^ (X), and N-terminus (R).

In the present study, we aim to develop NT(6–13) analogs by substituting Tyr^11^ with noncanonical amino acids. Many research groups have preferred to replace the amino acids adjacent to Tyr^11^ (i.e., Pro^10^ and Ile^12^) to address the instability of peptide bonds between Pro^10^-Tyr^11^ and Tyr^11^-Ile^12^, in part because Tyr^11^ residue plays a critical role in NTRS1 activation and signaling.^24,25^ For radiopharmaceuticals, receptor activation and signaling are not prerequisites for drug action, which is mediated by the radionuclide. We hypothesize that substituting Tyr^11^ would stabilize two peptide bonds concurrently, while allowing the reversion of Tle^12^ to native Ile^12^. The latter change is expected to improve the binding affinity for NTSR1.

## 2 Materials and Methods

### 2.1 General Methods

All chemicals and solvents were purchased from commercial suppliers and used without further purification. The NTSR1-targeting peptides were synthesized using a Liberty Blue automated microwave peptide synthesizer (CEM Corporation, Matthews, NC, USA). Purification of the DOTA-conjugated precursors was conducted on preparative Agilent HPLC system (Santa Clara, CA, USA) equipped with a model 1260 Infinity II preparative binary pump, a model 1260 Infinity variable wavelength detector (set at 220 nm), and a 1290 Infinity II preparative open-bed fraction collector. The system was operated with Agilent ChemStation software using a preparative column Luna C18 (Gemini, NX-C18, 5 μm, 110 Å, 50 × 30 mm; Phenomenex, Torrance, CA, USA). The nonradioactive standards were purified using a semipreparative Agilent HPLC system (Santa Clara, CA, USA) consisting of a model 1200 quaternary pump, a Luna C18 semipreparative column (Phenomenex, 5 μm, 100 Å, 250 × 10 mm), and a model 1200 UV absorbance detector (220 nm). Product-containing eluate fractions were collected, pooled, and lyophilized by using a Labconco FreeZone 4.5 Plus freeze-dryer (Kansas City, MO, USA). MS analyses of DOTA-conjugated precursors and their corresponding nonradioactive standards were performed using a Waters Acquity QDa mass spectrometer (Milford, MA, USA) equipped with a 2489 UV/vis detector and an e2695 Separations module. Gallium-68 was eluted from a generator purchased from ITM Medical Isotopes GmbH (Munich, Germany) and purified using a DGA resin column (Eichrom Technologies LLC, Lisle, IL, USA). ^68^Ga-labeled peptides were purified using an Agilent 1260 semipreparative HPLC system equipped with a model 1200 quaternary pump, a model 1200 UV detector set at 220 nm, and a NaI scintillation detector (Bioscan, Washington, DC, USA). The same semipreparative Luna C18 column was used for purification. The radioactivity of ^68^Ga-labeled peptides was measured using a CRC-25R/W dose calibrator (Capintec, Ramsey, NJ, USA). For biodistribution studies, the radioactivity in mouse tissues was quantified using either a Wizard2 2480 automatic gamma counter (PerkinElmer, Waltham, MA, USA) or a Hidex automatic gamma counter (Hidex, Turku, Finland).

### 2.2 Peptide Synthesis and Purification

#### 2.2.1 Synthesis of DOTA-Conjugated NT-20.3, SK01001, SK01003, SK01005, SM01010, SK01012, and SK01014 Precursors

The peptides were synthesized using solid-phase Fmoc chemistry, using the Liberty Blue automated peptide synthesizer. The synthesis was carried out using the standard procedure of Liberty Blue on the 0.1 mmol scale. The preloaded Fmoc-Leu-Wang resin (0.1 mmol, 333 mg) was swelled using 5 mL of *N,N*-dimethylformamide (DMF) for 5 min. The first deprotection of leucine was performed using 20% piperidine (v/v) in DMF for 2–3 min at 75 °C. The amino acids (0.1 mmol, 5 equiv) were sequentially coupled using *N,N′-*diisopropylcarbodiimide (5 equiv)/Oxyma Pure (5 equiv) in DMF at 95 °C for 2–3 min. The Fmoc deprotection of the last amino acid (Fmoc-l-lysine­(ivDde)) yielded a free N-terminal. Di-*tert*-butyl dicarbonate (20% v/v) in DMF was used to cap the free N-terminal, followed by deprotection of the ivDde group from the lysine side chain using hydrazine (2% v/v) in DMF. The DOTA­(tBu)_3_ (2 equiv), preactivated with HATU (2-(1H-7-azabenzotriazol-1-yl)-1,1,3,3-tetramethyl uranium hexafluorophosphate, methanaminium) (5 equiv) HOAt (1-hydroxy-7-azabenzotriazole) (5 equiv), and DIEA (*N,N*-diisopropylethylamine) (10 equiv), was coupled at the ε-NH_2_ of lysine. Whereas, in NT-20.3 and SK01014, the free N-terminus group was capped using acetic anhydride for overnight coupling. The final removal of protecting groups and cleavage from the resin was performed simultaneously using a mixture of trifluoroacetic acid (TFA, 81.5%), triisopropylsilane (TIS, 1.0%), water (5%), 2,2′(ethylenedioxy)­diethanethiol (DODT, 2.5%), thioanisole (5%), and phenol (5%) for 4 h at room temperature. After cleavage, the peptides were precipitated from the solution by using 30 mL of diethyl ether. The peptides were washed twice with diethyl ether and then dried under reduced pressure overnight. After complete drying, the crude products were dissolved in water, purified using preparative HPLC, and identified by using mass spectrometry. The purification conditions, retention times, isolated yields, and mass analyses of DOTA-conjugated precursors are provided in Supporting Information (refer to Table S1 and Figures S1–S7).

#### 2.2.2 Synthesis of ^nat^Ga-Complexed Standards

The peptide precursors (2 mg) were complexed with an excess of GaCl_3_ (10 equiv) in sodium acetate buffer (0.1 M, pH 4.5) at 95 °C for 15 min. The reaction solution was left to cool to ambient temperature, and ^nat^Ga complexation was confirmed by MS, followed by purification using semipreparative HPLC. Refer to the Supporting Information for HPLC conditions, retention times, and isolated yields (Table S2 and Figures S8–S14).

### 2.3 Cell Culture

The PC-3 prostate adenocarcinoma cell line (ATCC-CRL-1435) was maintained in Ham’s F-12K medium (Life Technologies, New York, NY, USA) supplemented with 10% fetal bovine serum (Sigma-Aldrich, New York, NY, USA), 100 IU/mL penicillin, and 100 μg/mL streptomycin (Life Technologies). Cells were cultured at 37 °C in a Panasonic Healthcare (Tokyo, Japan) MCO-19AIC humidified incubator containing 5% CO_2_. Cells were passaged at diluting ratios of 1:3 to 1:6, ensuring cells did not exceed 80–90% confluence. To harvest cells, they were washed with sterile Dulbecco’s phosphate-buffered saline (DPBS) and subsequently incubated with 0.25% Trypsin-EDTA solution for 1 min. Cell concentrations were measured in triplicate using a hemocytometer. The IMPACT Rodent Pathogen Test (IDEXX BioAnalytics, Columbia, MO, USA) was performed to confirm that the cell line was pathogen-free prior to animal experiments.

### 2.4 In Vitro Competitive Binding Assay

PC-3 cells were seeded at a density of 8 × 10^4^ cells per well in 24-well poly-d-lysine-coated plates and incubated at 37 °C for 48 h prior to the experiment. On the day of the assay, the culture medium was removed and replaced with 400 μL of reaction medium (F12K containing 2 mg/mL BSA and 20 mM HEPES), followed by a 1 h incubation at 37 °C. Subsequently, 50 μL of ^nat^Ga-labeled compounds (NT-20.3, SM01010, SK01001, SK01003, SK01005, SK01012, and SK01014) were added in triplicate across a concentration range from 10 μM to 1 pM. The radioligand [^125^I]­Tyr^3^-neurotensin (Revvity, Waltham, MA, USA) was added at a final concentration of 0.015 nM (50 μL per well). Plates were incubated at 25 °C with moderate orbital agitation. Following incubation, the medium was removed, cells were washed twice with 1 mL of DPBS, and harvested using 0.25% Trypsin-EDTA. Radioactivity was measured using a Hidex automated γ-counter. Data were analyzed using nonlinear regression (one-site binding model for competition assays) with GraphPad Prism software (version 10.1.2; GraphPad, San Diego, CA, USA).

### 2.5 Fluorometric Calcium Release Assay

The calcium release assay was performed following a previously described protocol.^30–32^ PC-3 cells were seeded at a density of 5 × 10^4^ cells per well in a clear-bottom 96-well plate and incubated for 24 h before the experiment. On the day of the assay, 100 μL of buffer containing a calcium-sensitive dye (FLIPR Calcium 6 Assay Kit, Molecular Devices, San Jose, CA, USA) was added to each well, followed by a 1-h incubation at 37 °C. The plate was then placed in a FlexStation 3 microplate reader (Molecular Devices, San Jose, CA, USA). ^Nat^Ga-complexed peptides, along with neurotensin (agonist control), SR 48692 (antagonist control), and DPBS (negative control), were added at a final concentration of 50 nM in triplicate. Fluorescence signals were recorded for 2 min using excitation at 485 nm and emission at 525 nm. Relative fluorescence units (RFUs) were used to evaluate the agonistic or antagonistic effects of the test compounds.

### 2.6 Cellular Internalization and Efflux Assay

For the internalization study, PC-3 cells were seeded at a density of 1 × 10^5^ cells per well in 24-well Poly-d-Lysine-coated plates and incubated for 48 h before the experiment. On the day of the assay, the growth medium was replaced with reaction buffer consisting of F12K medium supplemented with 20 mM HEPES and 2 mg/mL BSA, followed by a 1-h preincubation at 37 °C. Subsequently, ^68^Ga-labeled peptide (0.6–0.9 MBq) was added to each well and incubated at 37 °C for 15, 30, 60, and 90 min. Nonspecific binding was determined in parallel by coincubation with an excess (10 μM) of the neurotensin peptide. After incubation, the reaction buffer was removed and collected as supernatant fractions, and the cells were washed with ice-cold PBS, followed by two acid washes using acidic buffer (0.2 M acetic acid, 0.5 M NaCl, pH 2.5) for 10 min. The acid wash fractions were collected to quantify membrane-bound activity, while the remaining cells were harvested via trypsinization to determine the internalized fraction. All collected samples were analyzed for radioactivity using an automated gamma counter.

For the efflux study, PC-3 cells were incubated with ^68^Ga-labeled peptide (0.6–0.9 MBq) per well for 1 h at 37 °C to allow for internalization. Following incubation for 1 h, the reaction buffer was removed and replaced with fresh reaction buffer. Cells were then incubated further for 15, 30, 60, and 90 min at 37 °C to monitor peptide efflux. At each time point, the reaction buffer was collected to quantify released radioactivity, followed by washing the cells with ice-cold PBS. Cells were subsequently trypsinized to measure remaining membrane-bound and internalized radioactivity. All fractions were quantified using an automated gamma counter.

### 2.7 Radiolabeling and log*D*_7.4_ Measurements

Following elution of ^68^Ga from the ^68^Ge/^68^Ga generator, the isotope was purified using anion exchange chromatography, as previously described in the literature.^33^ The purified ^68^Ga (200–600 MBq) in 0.5 mL of deionized water was added to a reaction vial containing 0.7 mL of HEPES buffer (2 M, pH 5.0) and 10 nmol of precursor in water. The mixture was heated in a Monowave200 (Anton Paar, Graz, Austria) at 100 °C for 1 min. The radiolabeled product was then purified using semipreparative HPLC, and the collected radioactive fraction was diluted in 50 mL of water. This diluted solution was passed through a C18 Sep-Pak cartridge (preconditioned with 1 mL of ethanol, followed by 2 mL of water). The ^68^Ga-labeled compound was eluted from the cartridge with ethanol and subsequently diluted with 1 mL of PBS containing 1% ascorbic acid. The final formulation was used for PET imaging, biodistribution, and *in vivo* stability studies in mice. Quality control and molar activity were assessed using an analytical HPLC column (Luna C18, 5 μm, 250 × 4.6 mm) purchased from Phenomenex (Torrance, CA, USA). Detailed HPLC conditions and retention times are listed in Table S3. The octanol–water distribution coefficient was determined at pH 7.4 using the shake-flask method as reported previously.^30,33^ Briefly, aliquots (2 μL) of each radiotracer were added to a biphasic system consisting of 3 mL of *n*-octanol and 3 mL of PBS (pH 7.4) and vortexed for 2 min. Aliquots (1 mL) from each phase were collected into gamma counting tubes, and the radioactivity was measured using an automated gamma counter. The log*D*_7.4_ values of [^68^Ga]­Ga-NT-20.3, [^68^Ga]­Ga-SK01001, [^68^Ga]­Ga-SM01010, [^68^Ga]­Ga-SK01012, [^68^Ga]­Ga-SK01014 were calculated using the following equation.

$${\log D}_{7.4}{\mathrm{= log}}_{10}\mathrm{[(counts in octanol phase)/(counts in PBS buffer phase)].}$$

### 2.8 PET/CT Imaging and Biodistribution Studies

All animal experiments were conducted in accordance with the guidelines of the Canadian Council on Animal Care and were approved by the Animal Ethics Committee of the University of British Columbia (protocol #A20-0113). Male NOD.Cg-Rag1^tm1Mom^ Il2rg^tm1Wjl^/SzJ (NRG) mice, bred in-house, were obtained from the Animal Research Centre at BC Cancer Research Institute. PC-3 cells (5 × 10^6^) suspended in 100 μL of a 1:1 mixture of cell suspension and Matrigel were subcutaneously injected into the left shoulder of each mouse under anesthesia with 2.5% isoflurane in 2.0 mL/min oxygen. Once tumors reached a volume of 300–400 mm^3^, PET imaging and ex vivo biodistribution studies were carried out.

PET imaging studies of ^68^Ga-labeled tracers were conducted by using a Siemens (Knoxville, TN, USA) Inveon microPET/CT scanner. Approximately 3–4 MBq of ^68^Ga-labeled tracer was administered via the lateral caudal vein. A 10-min CT scan was first performed for anatomical localization and attenuation correction, followed by a 10-min static PET scan at 1 h postinjection. The body temperature of mice was maintained at 37 °C using thermal pads. PET data were acquired in list mode acquisition and reconstructed using the 3d-OSEM-MAP algorithm with CT-based attenuation correction. Three-dimensional regions of interest (ROIs) were placed on the reconstructed images to determine the %ID/g of tissue using the Inveon Acquisition Workplace software (conversion factor was predetermined using a ^68^Ge/^68^Ga source). For blocking studies, 100 μg of nonradioactive standard (Ga-SK01014) was coinjected with the radiotracer, and imaging was performed at 1-h postinjection. For biodistribution analysis, mice (*n* = 4–6 per group) were euthanized via CO_2_ inhalation, and blood was collected by cardiac puncture. Organs were excised, rinsed with PBS, blotted dry, weighed, and the associated radioactivity was measured using a PerkinElmer automated γ counter. Tissue uptake was expressed as the percentage of injected dose per gram of tissue (%ID/g).

### 2.9 In Vivo Stability Study

Healthy male NRG mice (*n* = 3 per group) were intravenously injected with [^68^Ga]­Ga-NT-20.3, [^68^Ga]­Ga-SK01012, or [^68^Ga]­Ga-SK01014 (∼6–7 MBq) via the lateral caudal vein. At 15 min postinjection, the mice were sedated and euthanized. Blood and urine samples were collected for analysis. To obtain plasma, 500 μL of blood was mixed with an equal volume of acetonitrile for protein precipitation, followed by centrifugation for 10 min. The resulting supernatant (plasma) and urine samples were filtered through a 0.22 μm syringe filter and subsequently analyzed by radio-HPLC under the same conditions used for quality control.

### 2.10 Statistical Analysis

The binding affinity values (*K*_i_) of Ga-SK01001, Ga-SM01010, Ga-SK01012, and Ga-SK01014 were compared with Ga-NT-20.3 using One-way ANOVA test followed by a Dunnet test. For calcium release studies, a two-tailed *t*-test was performed assuming equal variances using Microsoft Excel software. Biodistribution profile comparison between [^68^Ga]­Ga-NT-20.3 vs [^68^Ga]­Ga-SK01014 as well as between blocked and unblocked [^68^Ga]­Ga-SK01014 were performed using a multivariate unpaired *t*-test (Q = 1.00%) with GraphPad (version 10.1.2; GraphPad, San Diego, CA, USA). Prior to statistical comparison, outlier detection was performed using the ROUT method (*Q* = 1.00%)

## 3 Results

### 3.1 Peptide Synthesis, Radiolabeling, and Lipophilicity Measurements

The compounds NT-20.3, SK01001, SK01003, SK01005, SM01010, SK01012, and SK01014 were synthesized in yields ranging from 10% to 27%. The corresponding nonradioactive standards were obtained in 24% to 70% yields as shown in Supporting Information (Tables S1 and S2, Figures S1−S14). The synthesized nonradioactive standards were evaluated for their binding affinity toward NTSR1 (Figure 2 and Table 1). The summarized radiochemistry information and log*D*_7.4_ value are provided in Table 2. The radiolabeled products [^68^Ga]­Ga-NT-20.3, [^68^Ga]­Ga-SK01001, [^68^Ga]­Ga-SM01010, [^68^Ga]­Ga-SK01012, and [^68^Ga]­Ga-SK01014 were obtained in 22–60% decay-corrected isolated yield with molar activity >190 GBq/μmol and >95% radiochemical purity, as provided in Table 2. SK01003 and SK01005 were not radiolabeled because of poor binding affinity.

**Figure 2:**
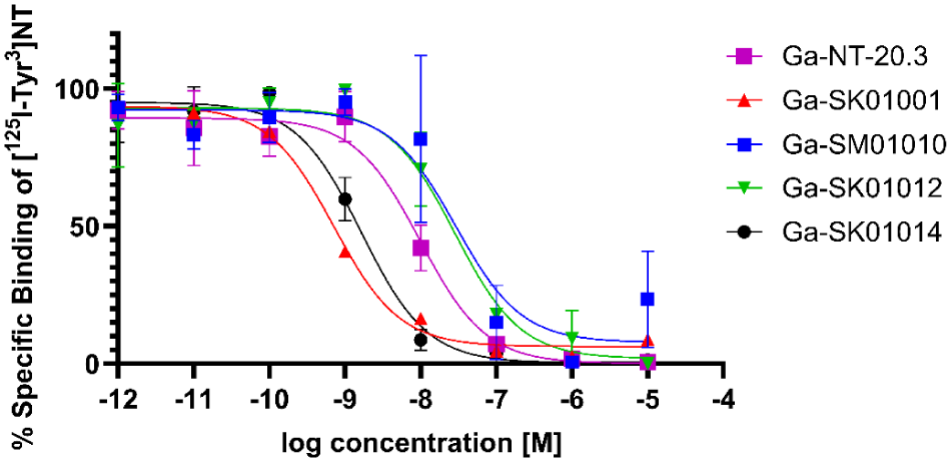
Displacement curves of Ga-NT-20.3, Ga-SK01001, Ga-SM01010, Ga-SK01012, and Ga-SK01014 from competition radioligand binding assays using an NTSR1-expressing PC-3 cell line and [^125^I-Tyr^3^]­NT as radioligand.

**Table 1:** Binding Affinity Values (*K*_i_) for ^nat^Ga-Labeled Analogs of NT-20.3, SK01001, SK01003, SK01005, SM01010, SK01012, and SK01014

Compound ID	Sequence	*K*_i_ (nM)(*n* = 3)
Ga-NT-20.3	Ac-Lys(DOTA)-Pro-NMeArg-Arg-Pro-Tyr-Tle-Leu	9.25 ± 3.37
Ga-SK01001	Lys(DOTA)-Pro-NMeArg-Arg-Pro-Dmt-Ile-Leu	0.60 ± 0.03
Ga-SK01003	Lys(DOTA)-Pro-NMeArg-Arg-Pro-Ala(9-anth)-Ile-Leu	>1000
Ga-SK01005	Lys(DOTA)-Pro-NMeArg-Arg-Pro-Phe(4-F)-Ile-Leu	86.5 ± 12.9
Ga-SM01010	Lys(DOTA)-Pro-NMeArg-Arg-Pro-1NaI-Ile-Leu	27.9 ± 16.5
Ga-SK01012	Lys(DOTA)-Pro-NMeArg-Arg-Pro-Dmt-Tle-Leu	25.0 ± 7.48
Ga-SK01014	Ac-Lys(DOTA)-Pro-NMeArg-Arg-Pro-Dmt-Ile-Leu	1.54 ± 0.26

**Table 2:** Radiolabeling and Log*D*_7.4_ Data of ^68^Ga-Labeled NT(6–13) Derivatives

Compound ID	Radiochemical Yield (%, decay-corrected)(*n* = 3)	Radiochemical Purity (%)(*n* = 3)	Log*D*_7.4_ value(*n* = 3)
[^68^Ga]Ga-NT-20.3	60.9 ± 3.08	>99	-2.43 ± 0.29
[^68^Ga]Ga-SK01001	43.1 ± 3.47	>98	-3.67 ± 0.04
[^68^Ga]Ga-SM01010	22.0 ± 2.96	>99	-2.55 ± 0.14
[^68^Ga]Ga-SK01012	51.8 ± 4.35	>97	-3.95 ± 0.21
[^68^Ga]Ga-SK01014	36.4 ± 11.7	>97	-3.04 ± 0.31

In terms of lipophilicity, the compounds ranked from least to most hydrophobic as follows: [^68^Ga]­Ga-SK01012, [^68^Ga]­Ga-SK01001, [^68^Ga]­Ga-SK01014, [^68^Ga]­Ga-SM01010, and [^68^Ga]­Ga-NT-20.3. [^68^Ga]­Ga-SM01010 exhibited similar lipophilicity as [^68^Ga]­Ga-NT-20.3, while [^68^Ga]­Ga-SK01001, [^68^Ga]­Ga-SK01012, and [^68^Ga]­Ga-SK01014 were more hydrophilic.

### 3.2 Binding Affinity and Agonist/Antagonist Characterization

The binding affinity values (*K*_i_) for Ga-NT-20.3, Ga-SK01001, Ga-SK01003, Ga-SK01005, Ga-SM01010, Ga-SK01012, and Ga-SK01014, along with their dose-dependent displacement of [1^25^I]­Tyr^3^-neurotensin confirming binding to NTSR1, are presented in Figure 2 and Table 1. Ga-SK01001 (0.60 ± 0.03 nM) and Ga-SK01014 (1.54 ± 0.26 nM) showed 15-fold (*p* < 0.01) and 6-fold (*p* < 0.05) improvements in binding affinity compared to Ga-NT-20.3 (9.25 ± 3.37 nM), respectively. The synthesized analogs with a *K_i_* of less than 30 nM were prioritized for further evaluation in preclinical studies, and their displacement curves are presented in Figure 2.

As shown in Figure 3, Ga-NT-20.3, Ga-SK01001, Ga-SM01010, Ga-SK01012, Ga-SK01014, and the agonist control (neurotensin peptide) significantly induced Ca^2+^ release with RFU values of 664 ± 116, 1000 ± 66.7, 503 ± 51.7, 660 ± 127, 607 ± 24.1, and 561 ± 130, respectively. On the other hand, antagonist control (SR 48692) and DPBS (buffer control) showed minimal Ca^2+^ release with RFU values of 8.93 ± 3.07 and 8.57 ± 1.20, respectively (*p* value <0.01).

**Figure 3:**
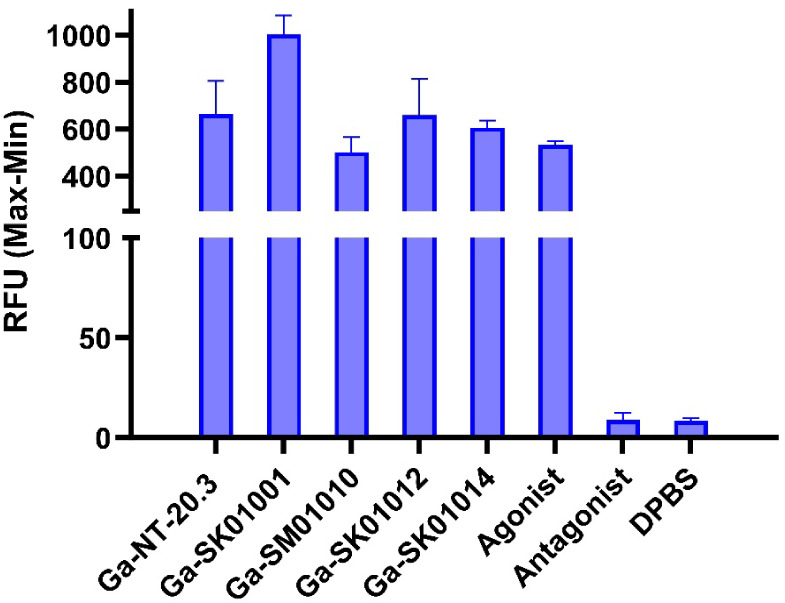
Induction of intracellular calcium release in PC-3 cells by Ga-NT-20.3, Ga-SK01001, Ga-SM01010, Ga-SK01012, and Ga-SK01014, along with the agonist control (Neurotensin), antagonist control (SR 48692), and DPBS.

### 3.3 Internalization and Efflux

Internalization of ^68^Ga-labeled peptides was investigated using the PC-3 cell line. [^68^Ga]­Ga-NT-20.3 showed rapid cellular uptake with 43.8 ± 3.91% internalization observed at 15 min, reaching a maximum of 46.4 ± 4.46% at 30 min. In contrast, [^68^Ga]­Ga-SK01014 exhibited a significantly high internalization rate, with 73.1 ± 3.04% at 15 min and reaching a maximum of 75.5 ± 2.40% at 30 min (*p* < 0.01 across all time points). The nonspecific cell uptake was effectively blocked by coincubation with an excess of neurotensin peptide at 90 min, confirming NTSR1-mediated internalization (Figure 4).

**Figure 4:**
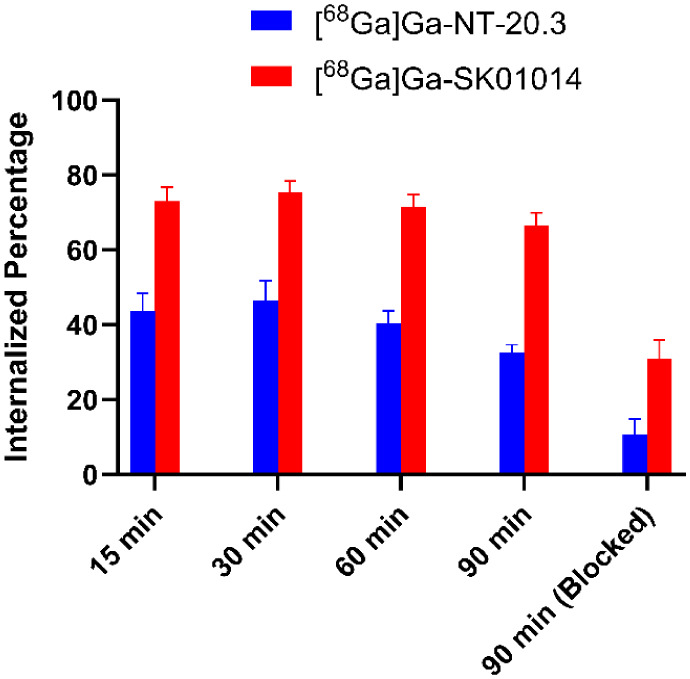
NTSR1-mediated internalization of [^68^Ga]­Ga-NT-20.3 and [^68^Ga]­Ga-SK01014 in PC-3 cells at 15, 30, 60, and 90 min (*n* = 3). Data are presented as Mean ± SD.

Efflux of the radiolabeled peptides was subsequently evaluated over the same time points. [^68^Ga]­Ga-NT-20.3 exhibited a gradual increase in efflux, reaching a maximum of 45.5 ± 1.31% at 90 min. In comparison, [^68^Ga]­Ga-SK01014 showed a lower extent of efflux, with a maximum of 27.0 ± 0.24% at 90 min. Notably, [^68^Ga]­Ga-SK01014 demonstrated significantly reduced efflux compared to [^68^Ga]­Ga-NT-20.3 across all time points (*p* < 0.05), as illustrated in Figure 5.

**Figure 5:**
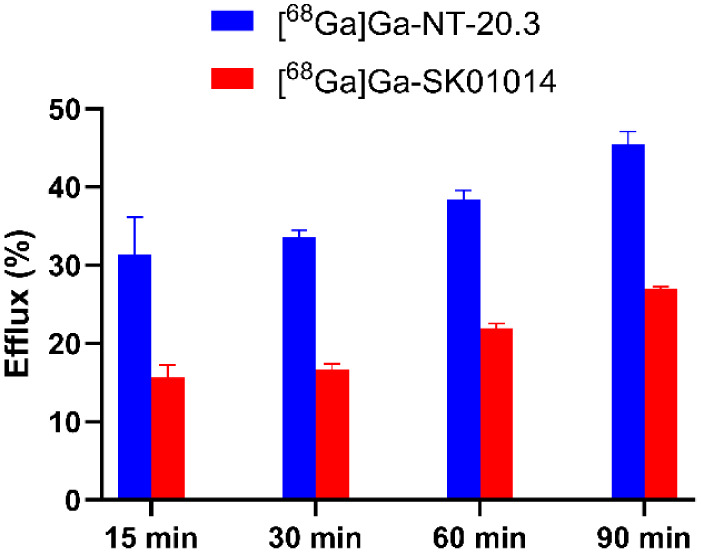
Efflux kinetics of [^68^Ga]­Ga-NT-20.3 and [^68^Ga]­Ga-SK01014 in PC-3 cells at 15, 30, 60, and 90 min (*n* = 3). Data are presented as the mean ± SD.

### 3.4 PET Imaging and Ex Vivo Biodistribution

Representative decay-corrected PET images of PC-3 tumor-bearing mice at 1 h postinjection are shown in Figure 6. [^68^Ga]­Ga-NT-20.3, [^68^Ga]­Ga-SK01001, [^68^Ga]­Ga-SM01010, [^68^Ga]­Ga-SK01012, and [^68^Ga]­Ga-SK01014 showed similar distribution profiles, with uptake observed in the PC-3 tumor xenograft, urinary bladder, kidneys, and, to a lesser extent, the intestines. The radiopharmaceuticals enabled clear visualization of the PC-3 tumor xenograft and were predominantly cleared through the kidneys. [^68^Ga]­Ga-SK01014 showed similar tumor uptake as [^68^Ga]­Ga-NT-20.3. Notably, the baseline image of [^68^Ga]­Ga-SK01014 had the lowest uptake or retention in the kidneys compared with all tested agents. The other agents, [^68^Ga]­Ga-SK01001, [^68^Ga]­Ga-SM01010, and [^68^Ga]­Ga-SK01012, had lower tumor uptake and tumor-to-background contrast than [^68^Ga]­Ga-NT-20.3. In the blocking study of [^68^Ga]­Ga-SK01014, where a nonradioactive standard (Ga-SK01014, 100 μg) was coinjected, the uptake in tumor and bowel was reduced to near background levels.

**Figure 6:**
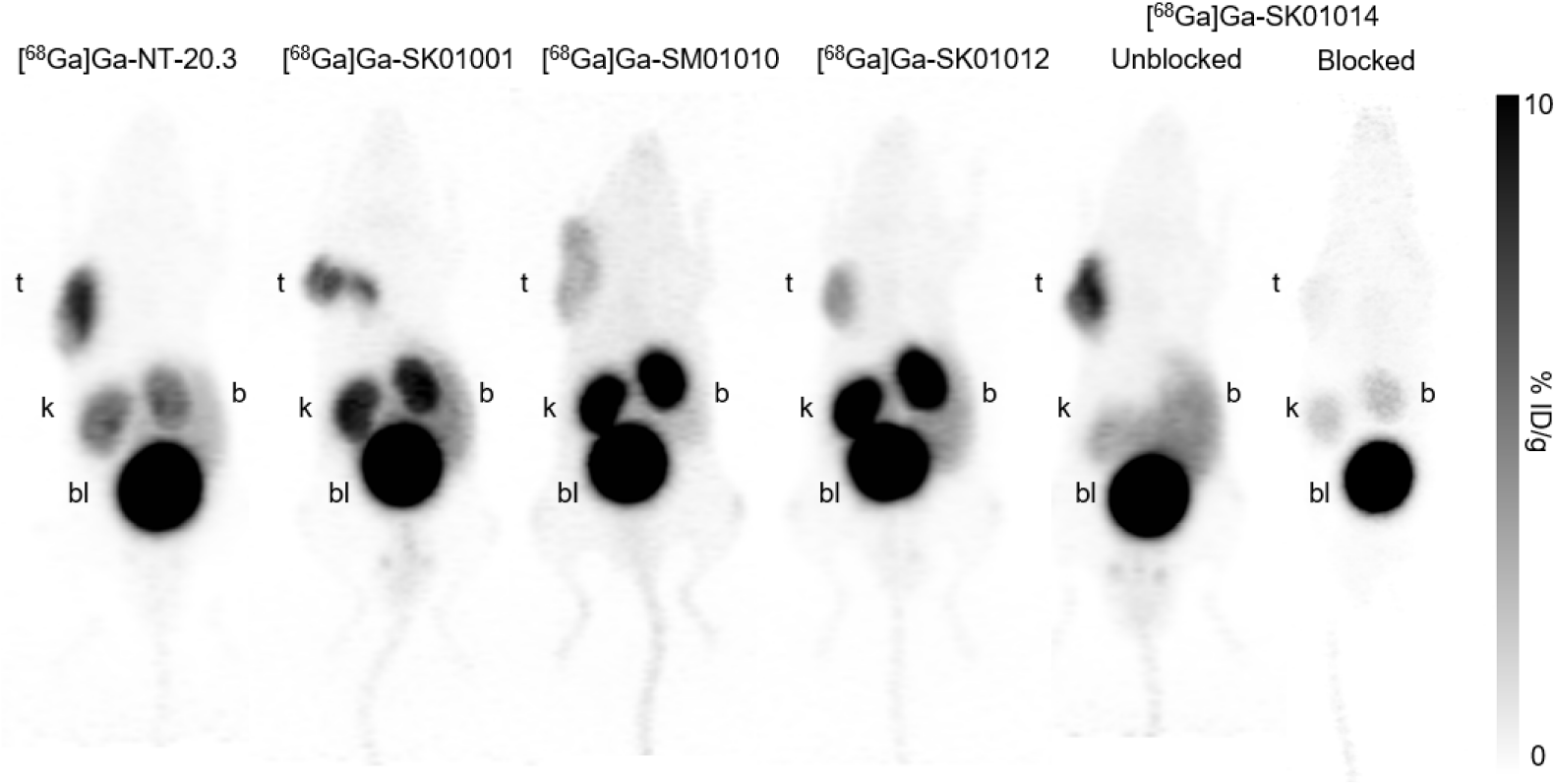
Representative PET images of [^68^Ga]­Ga-NT-20.3, [^68^Ga]­Ga-SK01001, [^68^Ga]­Ga-SM01010, [^68^Ga]­Ga-SK01012, and [^68^ Ga]­Ga-SK01014 acquired 1 h postinjection in PC-3 tumor-bearing mice. A blocking study was performed by coinjecting Ga-SK01014; t: tumor; k: kidney; bl: urinary bladder; b: bowel.

The *ex vivo* biodistribution studies for all five tracers were performed at 1 h postinjection (*n* = 4–6) and are presented in Table S4 and Figure 7. While [^68^Ga]­Ga-SK01014 showed comparable tumor uptake (10.0 ± 2.48%ID/g) compared to [^68^Ga]­Ga-NT-20.3 (9.43 ± 0.73%ID/g) (*p* value = 0.70), [^68^Ga]­Ga-SK01001, [^68^Ga]­Ga-SM01010, and [^68^Ga]­Ga-SK01012 exhibited lower tumor uptake values of 4.37 ± 0.96, 2.28 ± 0.45, and 5.88 ± 0.34%ID/g, respectively. For [^68^Ga]­Ga-SK01014, the kidney uptake value of 2.88 ± 0.64%ID/g was significantly lower than [^68^Ga]­Ga-NT-20.3 (9.66 ± 1.00%ID/g; *p* value <0.01), which resulted in a higher tumor-to-kidney ratio for [^68^Ga]­Ga-SK01014 of 3.53 than [^68^Ga]­Ga-NT-20.3 (0.99; *p* value <0.01). No significant difference in tumor-to-muscle ratio was observed between [^68^Ga]­Ga-SK01014 and [^68^Ga]­Ga-NT-20.3 (74.8 ± 41.6 vs 108 ± 44.1, *p* = 0.30), indicating that [^68^Ga]­Ga-SK01014 retains comparable target specificity to the reference compound. There was low to moderate uptake observed in the bowels, ranging from 1.09 to 6.09%ID/g, depending on the tracer. For other nontarget issues, the uptake in blood, fat, testes, stomach, spleen, pancreas, liver, heart, lungs, brain, muscle, and bone was <1%ID/g. Figure 8 presents the results of blocking studies for [^68^Ga]­Ga-SK01014. The coinjection of the blocking agent significantly reduced tumor uptake by 92.7% (from 10.0 ± 2.48%ID/g to 0.73 ± 0.22%ID/g) (*p* < 0.01), small intestine by 94.5% (from 6.09 ± 0.99%ID/g to 0.31 ± 0.10%ID/g) (*p* < 0.01), and large intestine by 93.5% (from 2.17 ± 0.75%ID/g to 0.14 ± 0.06%ID/g) (*p* < 0.01), respectively.

**Figure 7:**
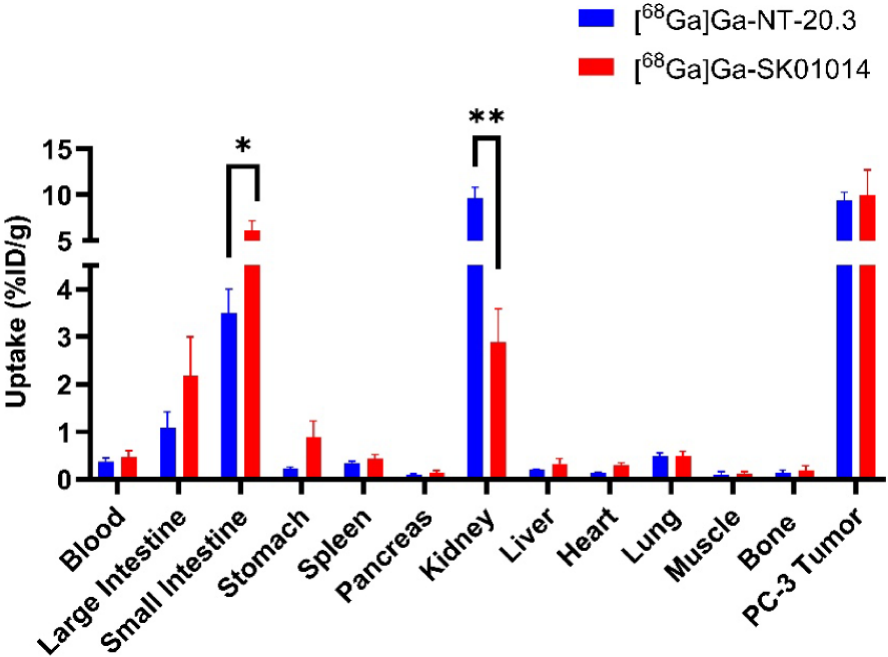
Comparison of biodistribution profile of major tissues/organs for [^68^Ga]­Ga-NT-20.3 and [^68^Ga]­Ga-SK01014 acquired 1 h postinjection in PC-3 tumor-bearing mice, *p < 0.05, **p < 0.01.

**Figure 8:**
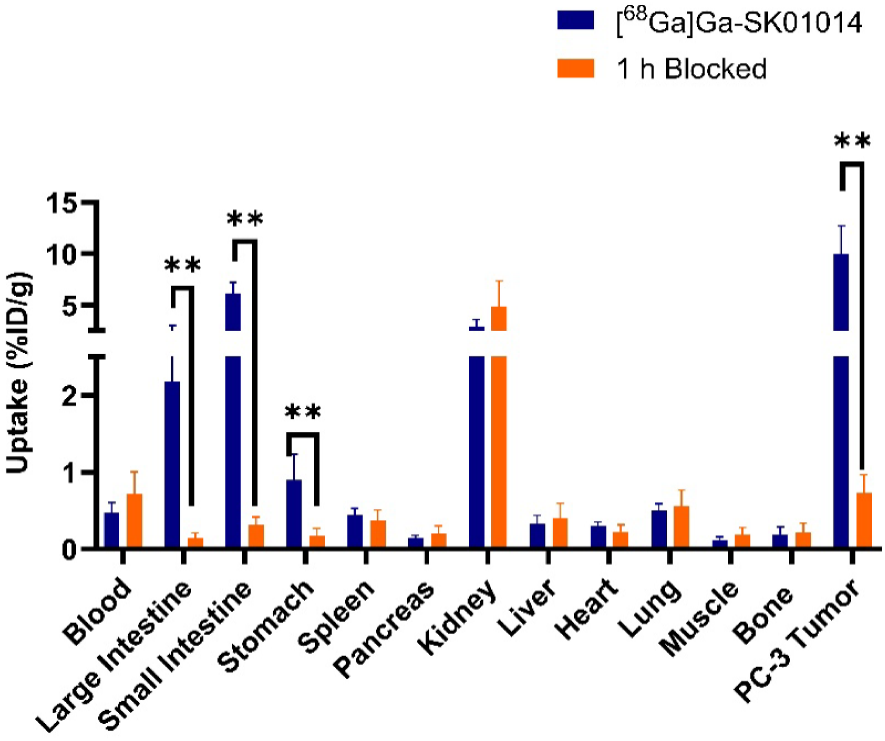
Comparison of biodistribution profile of major tissues/organs for [^68^Ga]­Ga-SK01014 with and without coinjection of 100 μg of nonradioactive standard (Ga-SK01014) acquired 1 h postinjection in PC-3 tumor-bearing mice, **p < 0.01.

### 3.5 In Vivo Stability

The *in vivo* stability was performed by injecting ^68^Ga-labeled peptides in healthy male mice (*n* = 3) at 15 min postinjection, collecting plasma and urine, and performing radio-HPLC analysis (Figures S15–S17). The percentages of intact radiotracers found in plasma and urine are shown in Table 3. [^68^Ga]­Ga-NT-20.3 and [^68^Ga]­Ga-SK01012 showed comparable stability, with 64% and 79% remaining intact in plasma, respectively. In contrast, the intact fraction of [^68^Ga]­Ga-SK01014 was only 34%, indicating plasma degradation. The radio-HPLC chromatograms of plasma and urine samples are provided in the Supporting Information (Figures S15–S17).

**Table 3:** % Remaining Intact Fractions of [^68^Ga]­Ga-NT-20.3, [^68^Ga]­Ga-SK01012, and [^68^Ga]­Ga-SK01014 in Mouse Plasma and Urine After 15 Min Postinjection (N = 3)

Compound ID	% Remaining Intact Fraction in Plasma	% Remaining Intact Fraction in Urine
[^68^Ga]Ga-NT-20.3	64.1 ± 5.91	84.2 ± 2.60
[^68^Ga]Ga-SK01012	78.9 ± 9.67	85.9 ± 2.53
[^68^Ga]Ga-SK01014	34.0 ± 5.53	44.2 ± 7.88

## 4 Discussion

NTSR1 has long been considered a promising theranostic target for solid malignancies, including but not limited to colorectal cancer, gastrointestinal cancer, and PDAC. These cancers are extremely difficult to treat, especially in metastatic settings. As with many GPCR systems (e.g., gastrin-releasing peptide receptor,^34,35^ cholecystokinin type 2 receptor,^36,37^ and bradykinin B1 receptor,^38^ etc.), modifying the endogenous ligand to serve as the targeting vector is a common design strategy. This approach requires balancing different drug parameters, including binding affinity, lipophilicity, *in vivo* stability, and bioavailability. Another emergent approach for targeting NTRS1 is to use nonpeptidic antagonists as pharmacophores.^39–41^ Baum et al. published on the compassionate use of [^177^Lu]­Lu-3BP-227 in six patients with metastatic PDAC who had exhausted conventional treatment.^42^ A terminal patient with ascites formation achieved partial remission, surviving for 11 months following treatment. However, care should be exercised when interpreting the data given the retrospective nature of the study, the heterogeneous patient population, and the concomitant chemotherapies received by patients. An actinium-225 version of [^177^Lu]­Lu-3BP-227, [^225^Ac]­Ac-FPI-2059, was recently advanced to a phase I trial in patients with solid tumors (NCT05605522).^43^

NT analog stability is typically enhanced through backbone modifications that limit protease susceptibility, including replacement of labile peptide bonds with reduced amide isosteres, incorporation of unnatural amino acids, and N-terminal capping. These strategies are frequently combined with macrocyclization or selective side-chain alterations to markedly prolong plasma half-life for imaging or therapeutic applications.^5,23,44–49^ The substitution of Ile^12^ with Tle^12^ is a well-established approach to stabilize the Tyr^11^-Ile^12^ peptide bond, as in the case of [^68^Ga]­Ga-NT-20.3. In the present study, we substituted Tyr^11^ with various aromatic noncanonical amino acids to enhance binding affinity and stabilize the Pro^10^–Tyr^11^ and Tyr^11^–Ile^12^ cleavage sites. Based on structure–activity relationship studies,^50–52^ the presence of a π-electron system at position 11 is essential for binding to NTSR1. Specifically, we introduced 9-anthryl-alanine (Ala­(9-anth)) in SK01003, 4-fluorophenylalanine (Phe­(4-F)) in SK01005, 1-naphthylalanine (1-Nal) in SM01010, and 2,6-dimethyltyrosine (Dmt) in both SK01001 and SK01014. Consistent with NT-20.3, the Arg^8^ residue was modified to NMeArg. However, Tle^12^ was reverted to native Ile^12^. In contrast, the SK01012 compound was synthesized with dual modifications: replacement of Tyr^11^ with Dmt^11^ and Ile^12^ with Tle^12^.

Based on cell competition assays, the compounds with the strongest binding affinities (*K*_i_ < 2 nM) were Ga-SK01001 and Ga-SK01014, both containing Dmt^11^ substitutions, representing approximately 14-fold and 6-fold improvements in *K*_i_, respectively, compared with Ga-NT-20.3. Ga-SK01001 and Ga-SK01014 share an identical peptide sequence, with the sole difference being that Ga-SK01014 is N-terminally acetylated. However, the Tyr^11^ to Dmt^11^ modification did not universally improve the binding affinity across all compounds. Despite containing a Dmt^11^ substitution, Ga-SK01012, which also incorporates a Tle^12^, showed reduced binding affinity (*K*_i_ = 25.0 nM), likely attributable to the Ile^12^-to-Tle^12^ substitution. This finding supports the importance of reverting to Ile^12^ for achieving a high NTSR1 binding affinity for these agents. By evaluating 1-Nal and Ala­(9-anth), we were able to study the impact of aromatic ring size on binding affinity. Ga-SM01010, containing 1-Nal, exhibited a *K*_i_ value of 28 nM, whereas Ga-SK01003, containing Ala­(9-anth), showed a *K*_i_ value of >1000 nM. The results suggest that the Tyr^11^ position can accommodate aromatics up to a bicyclic ring like naphthalene but not a tricyclic ring like anthracene, likely due to steric constraints. We predicted that Ga-SK01005, containing Phe­(4-F)^11^ and Ile^12^ substitutions, would display similar or improved binding affinity relative to Ga-NT-20.3, as Cusask et al. previously reported that [F-Phe]­NT(8–13) exhibited IC_50_ value in the low nanomolar range.^52^ Contrary to this expectation, SK01005 showed a *K*_i_ of 86.5 nM, suggesting that the phenolic hydroxyl group of Tyr^11^ plays a critical role in forming hydrophilic interactions within the NTSR1 binding pocket.

Ga-NT-20.3, Ga-SM01010, Ga-SK01001, and Ga-SK01014 were all characterized as agonists based on calcium release assays, in which each compound induced Ca^2+^ influx comparable to that observed with the agonist control. In the context of GPCR-targeting radiopharmaceuticals, both agonists and antagonists can serve as effective pharmacophores.^53,54^ NTSR1, as a member of the GPCR family, is known to undergo rapid and significant internalization primarily via a β-arrestin-dependent pathway upon agonist binding. In this study, [^68^Ga]­Ga-SK01014 showed rapid and higher internalization (75.5 ± 2.40% at 30 min) upon binding to NTSR1 compared to [^68^Ga]­Ga-NT-20.3 (46.4 ± 4.46% at 30 min). The enhanced internalization observed for [^68^Ga]­Ga-SK01014 is likely attributed to its higher binding affinity for NTSR1. Ligand internalization does not necessarily guarantee prolonged tumor retention, as rapid recycling can lead to accelerated washout. As recently highlighted by Bodin et al., efflux kinetics may ultimately have a greater influence on imaging contrast and therapeutic efficacy than conventional parameters (e.g., binding affinity) for NTSR1 radiopharmaceuticals.^44^ Given that all evaluated compounds function as agonists, the rate of ligand efflux becomes a critical parameter to consider. [^68^Ga]­Ga-NT-20.3 demonstrated significantly higher efflux (45.5 ± 1.31%) compared to [^68^Ga]­Ga-SK01014, which showed only 27.0 ± 0.24% efflux at 90 min, suggesting cellular retention of the latter.

As an internal selection threshold, peptides exhibiting *K*_i_ values of <30 nM were advanced into preclinical evaluation. [^68^Ga]­Ga-SK01001, [^68^Ga]­Ga-SM01010, [^68^Ga]­Ga-SK01012, and [^68^Ga]­Ga-SK01014 were radiolabeled following standard literature procedures. Although the DOTA chelator enables coordination of a broad range of radionuclides, gallium-68 was selected for two primary reasons: first, its physical half-life (67.7 min) is well matched to the expected pharmacokinetics of NT analogs, and second, it allows direct comparison with reference [^68^Ga]­Ga-NT-20.3. For preclinical evaluation of NTSR1-targeted imaging agents, commonly used cancer models include PC-3 prostate adenocarcinoma, AsPC-1 pancreatic adenocarcinoma, and HT-29 colorectal adenocarcinoma.^14,55^ Based on comparable uptake of [^68^Ga]­Ga-NT-20.3, these three models appear to express similar levels of NTSR1. In the present study, the PC-3 xenograft model was selected for an *in vivo* evaluation. The PET images were consistent with the *ex vivo* biodistribution data obtained at 1 h postinjection. The tested tracers exhibited clean distribution profiles, with uptake predominantly observed in the PC-3 tumor xenograft, urinary bladder, kidneys, and, to a lesser extent, the intestines. Renal and bladder activity is consistent with urinary excretion, while the uptake in the tumor and intestines reflects NTSR1-expressing tissues.^56–59^ In mice coinjected with a blocking agent, both tumor and intestinal uptake were reduced to near-background levels, demonstrating tracer specificity. [^68^Ga]­Ga-SK01001, which showed high binding affinity *in vitro*, showed moderate tumor and kidney uptake of 4.37 ± 0.96 and 11.1 ± 1.60%ID/g, respectively. Interestingly, [^68^Ga]­Ga-SK01012, bearing both the Dmt and Tle substitutions, showed comparable tumor uptake (5.88 ± 0.34%ID/g) to [^68^Ga]­Ga-SK01001. The highest tumor uptake was observed with [^68^Ga]­Ga-SK01014 (10.0 ± 2.48%ID/g), which is comparable to [^68^Ga]­Ga-NT-20.3 (9.43 ± 0.73%ID/g), but with significantly reduced kidney uptake (2.88 ± 0.64 vs 9.66 ± 1.00%ID/g, respectively). Among all of the compounds tested, [^68^Ga]­Ga-SK01014 demonstrated the lowest renal accumulation. This reduction is attributed to N-terminal acetylation, which neutralizes the positive charge of the α-amine on Lys^6^ in NT(6–13) analogs. Positively charged peptides are known to undergo increased renal reabsorption via cationic transporter mechanisms.^60^ Importantly, the ability to modulate renal uptake without compromising tumor accumulation is advantageous for mitigating potential nephrotoxicity, a common concern associated with radioligand therapies based on radiolabeled peptides.

We hypothesized that substitution at Tyr^11^ would stabilize two labile peptide bonds within the NT(6–13) sequence; however, *in vivo* stability at 15 min postinjection revealed divergent metabolic profiles that did not directly correlate with tumor uptake. [^68^Ga]­Ga-SK01012, incorporating Dmt^11^ and Tle^12^, exhibited the highest plasma stability (78.9% intact), yet demonstrated reduced tumor accumulation compared with [^68^Ga]­Ga-NT-20.3 and [^68^Ga]­Ga-SK01014. This is likely attributed to its comparatively lower binding affinity, indicating that enhanced metabolic stability alone is insufficient to ensure improved tumor targeting. In contrast, [^68^Ga]­Ga-SK01014 underwent pronounced metabolic degradation in both plasma (34.0% intact) and urine (44.2% intact). This likely reflected continued recognition of the Dmt^11^-Ile^12^ motif by neprilysin, as substitution at Tyr^11^ may be insufficient to fully abrogate the enzyme’s access when paired with Ile^12^. Despite reduced stability, ^68^Ga]­Ga-SK01014 achieved comparable tumor uptake to [^68^Ga]­Ga-NT-20.3, suggesting that favorable NTSR1 binding affinity, rapid internalization, and reduced efflux can partially compensate for rapid *in vivo* degradation at early imaging time points. These findings highlight the trade-off between metabolic stability and NTSR1 binding affinity, emphasizing the difficulty of improving resistance to neprilysin-mediated cleavage without perturbing receptor engagement.

A major limitation of this study is that imaging was performed at a single early time point (1 h postinjection), without assessment at later time points. For [^68^Ga]­Ga-NT-20.3, prior preclinical studies have shown that tumor retention decreases by approximately 50% at later times, raising the possibility that alternative analogs such as [^68^Ga]­Ga-SK01014 could exhibit better tumor uptake or tumor-to-background contrast at delayed imaging. However, given the pronounced metabolic instability observed for [^68^Ga]­Ga-SK01014, we elected not to pursue extended imaging studies and instead focused on alternative optimization strategies. Our current efforts prioritize retention of the Dmt^11^-Ile^12^ modification while systematically exploring linker composition, N-terminal modifications, and C-terminal optimization to better balance metabolic stability and NTSR1 binding affinity.

## 5 Conclusion

In this study, a series of NT(6–13) analogs was synthesized by substituting the Tyr^11^ position with unnatural aromatic amino acids and systematically evaluated for NTSR1 binding affinity. Compounds exhibiting *K*_i_ values <30 nM were advanced to preclinical evaluation. The selected analogs (Ga-SK01001, Ga-SM01010, Ga-SK01012, and Ga-SK01014) all retained agonist activity. Among the substitutions tested, Dmt consistently provided superior in vitro binding affinity compared with other bulky aromatic residues, including Ala­(9-anth) and 1-Nal, with SK01001 displaying subnanomolar affinity. The combination of Dmt substitution and N-terminal acetylation in [^68^Ga]­Ga-SK01014 resulted in nanomolar-range binding affinity, high internalization capacity, reduced efflux, comparable tumor uptake, and significantly enhanced renal clearance compared to [^68^Ga]­Ga-NT-20.3, leading to an improved tumor-to-kidney ratio. However, the observed *in vivo* degradation of [^68^Ga]­Ga-SK01014 indicates the need for further improvements in metabolic stability.

## Supplementary Material



## Notes

Additional data of HPLC purification and Radio-HPLC purification conditions, MS spectra, *In vivo* stability chromatograms (PDF)

## Abbreviations

NT: neurotensin
NTSR1: neurotensin receptor 1
NTSR2: neurotensin receptor 2
GPCR: G protein-coupled receptor
Leu: l-leucine
Ile: l-isoleucine
Tyr: l-tyrosine
Pro: l-proline
Lys: l-lysine
Arg: l-arginine
DOTA: 1,4,7,10-tetraazacyclododecane-1,4,7,10-tetraacetic acid
Ala­(9-anth): 9-anthryl-alanine
Phe­(4-F): 4-fluorophenylalanine
1-Nal: 1-naphthylalanine
Dmt: 2,6-dimethyltyrosine
Tle: *tert*-leucine
%ID/g: percentage of the injected dose per gram of tissue
PDAC: pancreatic ductal adenocarcinoma
PET: positron emission tomography
HPLC: high-performance liquid chromatography
MS: mass spectrometry
ATCC: American Type Culture Collection
IMPACT: infectious microbe PCR amplification test
HEPES: 2-[4-(2-hydroxyethyl)­piperazin-1-yl]­ethanesulfonic acid
BSA: bovine serum albumin
DPBS: Dulbecco’s phosphate-buffered saline
NRG: NOD.Cg-Rag1^tm1Mom^ Il2rg^tm1Wjl^/SzJ
RFU: relative fluorescent unit
CT: computed tomography
PC-3: human prostate adenocarcinoma cell line
