# Supplementary material for: Synthesis and Evaluation of 68Ga-Labeled NT(6–13) Analogs Incorporating Non-Canonical Amino Acid Substitutions at Tyr11 for Targeting NTSR1 in Various Solid Malignancies
Source: ACS Omega. 2026 Apr 29;11(18):27535–45. doi: 10.1021/acsomega.6c02356 (PMC13206671; doi:10.1021/acsomega.6c02356)
Supplement: Supplementary file 1 [file ao6c02356_si_001.pdf]

Supplemental Information

Synthesis and Evaluation of ^{68}Ga-Labeled NT(6-13) Analogs Incorporating Non-Canonical Amino Acid Substitutions at Tyr^{11} for Targeting NTSR1 in Various Solid Malignancies

Simranjeet Kaur[1,2]*, Stefan Mair*[1]*, Wing Sum Lau*[1]*, Jutta Zeisler*[1,2]*, François Bénard*[1,2]*, Kuo-Shyan Lin*[1,2]*, Joseph Lau*[1,2*]

[1]Department of Basic and Translational Research, BC Cancer Research Institute, Vancouver, BC V5Z1L3, Canada;

[2]Department of Radiology, University of British Columbia, Vancouver, BC V5Z1M9, Canada

[*]Correspondence: jlau@bccrc.ca; 1-604-675-8026

Table S1: Purification conditions, retention times, yields (%) and mass confirmations of DOTA-conjugated precursors

Compound ID	HPLC conditions	Retention times (min)	Yield (%)	Calculated $[M+2H]^{2+}$ (*m/z*)	Observed $[M+2H]^{2+}$ (*m/z*)
NT-20.3	15-30% ACN (0.1%TFA) in H_2O in 15 min; Flow rate: 30 mL/min	5.71	11.7	742.92	743.17
SK01001	17-24% ACN (0.1%TFA) in H_2O in 14 min; Flow rate: 30 mL/min	6.47	10.9	735.93	736.12
SK01003	15-35% ACN (0.1%TFA) in H_2O in 20 min; Flow rate: 30 mL/min	13.90	20.0	763.94	764.77
SK01005	15-25% ACN (0.1%TFA) in H_2O in 6 min, then 25% ACN (0.1%TFA) in H_2O isocratic for 6 min; Flow rate: 30 mL/min	5.50	16.7	722.92	723.15
SM01010	15-30% ACN (0.1%TFA) in H_2O in 15 min; Flow rate: 30 mL/min	10.50	27.6	738.93	739.28
SK01012	15-20% ACN (0.1%TFA) in H_2O in 15 min; Flow rate: 30 mL/min	8.80	13.3	735.93	736.21
SK01014	15-20% ACN (0.1%TFA) in H_2O in 15 min; Flow rate: 30 mL/min	12.30	10.2	756.94	757.25

Table S2: Purification conditions, retention times, yields (%) and mass confirmations of ^{nat}Ga-complexed standards

Compound ID	HPLC conditions	Retention times (min)	Yield (%)	Calculated $[M+2H]^{2+}$ (*m/z)*	Observed $[M+2H]^{2+}$ (*m/z*)
Ga-NT-20.3	20% ACN (0.1%TFA) in H_2O; Flow rate: 4.5 mL/min	12.90	40.9	776.38	776.70
Ga-SK01001	23% ACN (0.1%TFA) in H_2O; Flow rate: 4.5 mL/min	10.75	47.9	769.39	769.10
Ga-SK01003	29% ACN (0.1%TFA) in H_2O; Flow rate: 4.5 mL/min	11.95	26.4	796.89	797.68
Ga-SK01005	21% ACN (0.1%TFA) in H_2O; Flow rate: 4.5 mL/min	22.90	24.4	765.35	765.23
Ga-SM01010	26% ACN (0.1%TFA) in H_2O; Flow rate: 4.5 mL/min	8.12	44.8	772.38	772.77
Ga-SK01012	21% ACN (0.1%TFA) in H_2O; Flow rate: 4.5 mL/min	11.70	36.3	769.39	769.27
Ga-SK01014	24% ACN (0.1%TFA) in H_2O; Flow rate: 4.5 mL/min	8.00	70.8	789.89	790.35

Table S3: Quality Control of ^{68}Ga-labeled peptides; HPLC conditions and retention times

Compound ID	HPLC conditions	Retention times (min)
[^{68}Ga]Ga-NT-20.3	21% ACN (0.1%TFA) in H_2O Flow rate: 2 mL/min	8.80
[^{68}Ga]Ga-SM01010	27% ACN (0.1%TFA) in H_2O Flow rate: 2 mL/min	10.50
[^{68}Ga]Ga-SK01001	22% ACN (0.1%TFA) in H_2O Flow rate: 2 mL/min	7.08
[^{68}Ga]Ga-SK01012	21% ACN (0.1%TFA) in H_2O Flow rate: 2 mL/min	8.05
[^{68}Ga]Ga-SK01014	23% ACN (0.1%TFA) in H_2O Flow rate: 2 mL/min	7.10

Table S4. Complete biodistribution profile of [^{68}Ga]Ga-NT-20.3, [^{68}Ga]Ga-SK01001, [^{68}Ga]Ga-SM01010, [^{68}Ga]Ga-SK01012, and [^{68}Ga]Ga-SK01014 (with and without blocking) in PC-3 tumor xenograft bearing mice at 1h post-injection (n = 4-6), $^{**}p < 0.01$.

Compound ID	[^{68}Ga]Ga-NT-20.3	[^{68}Ga]Ga-SK01001	[^{68}Ga]Ga-SM01010	[^{68}Ga]Ga-SK01012	[^{68}Ga]Ga-SK01014	
Tissue/Organs	Baseline (n = 4)	Baseline (n = 4)	Baseline (n = 5)	Baseline (n = 4)	Baseline (n = 6)	Blocked (n = 5)
Blood	0.37 ± 0.07	0.39 ± 0.20	0.52 ± 0.12	0.48 ± 0.11	0.47 ± 0.12	0.72 ± 0.26
Fat	0.06 ± 0.01	0.05 ± 0.02	0.09 ± 0.03	0.28 ± 0.22	0.08 ± 0.02	0.10 ± 0.04
Testes	0.11 ± 0.02	0.12 ± 0.04	0.19 ± 0.05	0.21 ± 0.05	0.15 ± 0.04	0.17 ± 0.09
Large intestine	1.09 ± 0.29	1.01 ± 0.18	1.14 ± 0.13	1.39 ± 0.24	2.17 ± 0.75	0.14 ± 0.06**
Small intestine	3.50 ± 0.43	3.25 ± 0.81	2.47 ± 0.22	4.29 ± 0.33	6.09 ± 0.99	0.31 ± 0.10**
Stomach	0.22 ± 0.03	0.40 ± 0.14	0.47 ± 0.11	0.46 ± 0.26	0.90 ± 0.30	0.17 ± 0.09**
Spleen	0.34 ± 0.04	0.27 ± 0.06	0.45 ± 0.04	0.38 ± 0.04	0.44 ± 0.08	0.37 ± 0.12
Pancreas	0.11 ± 0.01	0.09 ± 0.03	0.40 ± 0.27	0.15 ± 0.02	0.15 ± 0.03	0.20 ± 0.09
Adrenal glands	0.48 ± 0.21	0.99 ± 0.39	0.50 ± 0.08	0.76 ± 0.27	1.04 ± 0.51	0.32 ± 0.17
Kidneys	9.66 ± 1.00	11.1 ± 1.59	20.6 ± 2.05	24.7 ± 6.03	2.88 ± 0.64	4.86 ± 2.24
Liver	0.20 ± 0.01	0.24 ± 0.08	0.42 ± 0.08	0.38 ± 0.06	0.33 ± 0.10	0.40 ± 0.17
Heart	0.13 ± 0.01	0.20 ± 0.05	0.21 ± 0.04	0.26 ± 0.02	0.30 ± 0.04	0.22 ± 0.08
Lung	0.49 ± 0.06	0.30 ± 0.10	0.52 ± 0.09	0.61 ± 0.06	0.50 ± 0.08	0.56 ± 0.19
Brain	0.02 ± 0.00	0.02 ± 0.00	0.03 ± 0.00	0.02 ± 0.01	0.03 ± 0.01	0.03 ± 0.01
Muscle	0.08 ± 0.05	0.07 ± 0.02	0.18 ± 0.00	0.11 ± 0.02	0.12 ± 0.04	0.18 ± 0.09
Bone	0.15 ± 0.04	0.09 ± 0.02	0.38 ± 0.36	0.14 ± 0.02	0.19 ± 0.09	0.22 ± 0.11
PC-3 tumor	9.43 ± 0.73	4.37 ± 0.96	2.29 ± 0.45	5.88 ± 0.34	10.0 ± 2.48	0.73 ± 0.22**
Tumor-to-Normal Tissue Ratio						
Tumor/blood	25.7 ± 3.38	13.8 ± 5.56	4.59 ± 1.12	13.1 ± 3.29	21.7 ± 6.19	1.03 ± 0.07
Tumor/muscle	108 ± 44.1	85.8 ± 31.3	19.8 ± 13.6	53.9 ± 9.88	74.8 ± 41.6	2.30 ± 2.10
Tumor/kidney	0.99 ± 0.15	0.39 ± 0.06	0.11 ± 0.02	0.25 ± 0.07	3.53 ± 0.84	1.86 ± 0.04
Tumor/liver	47.0 ± 1.65	18.9 ± 4.24	5.54 ± 0.94	15.7 ± 1.53	32.2 ± 10.2	1.05 ± 0.40

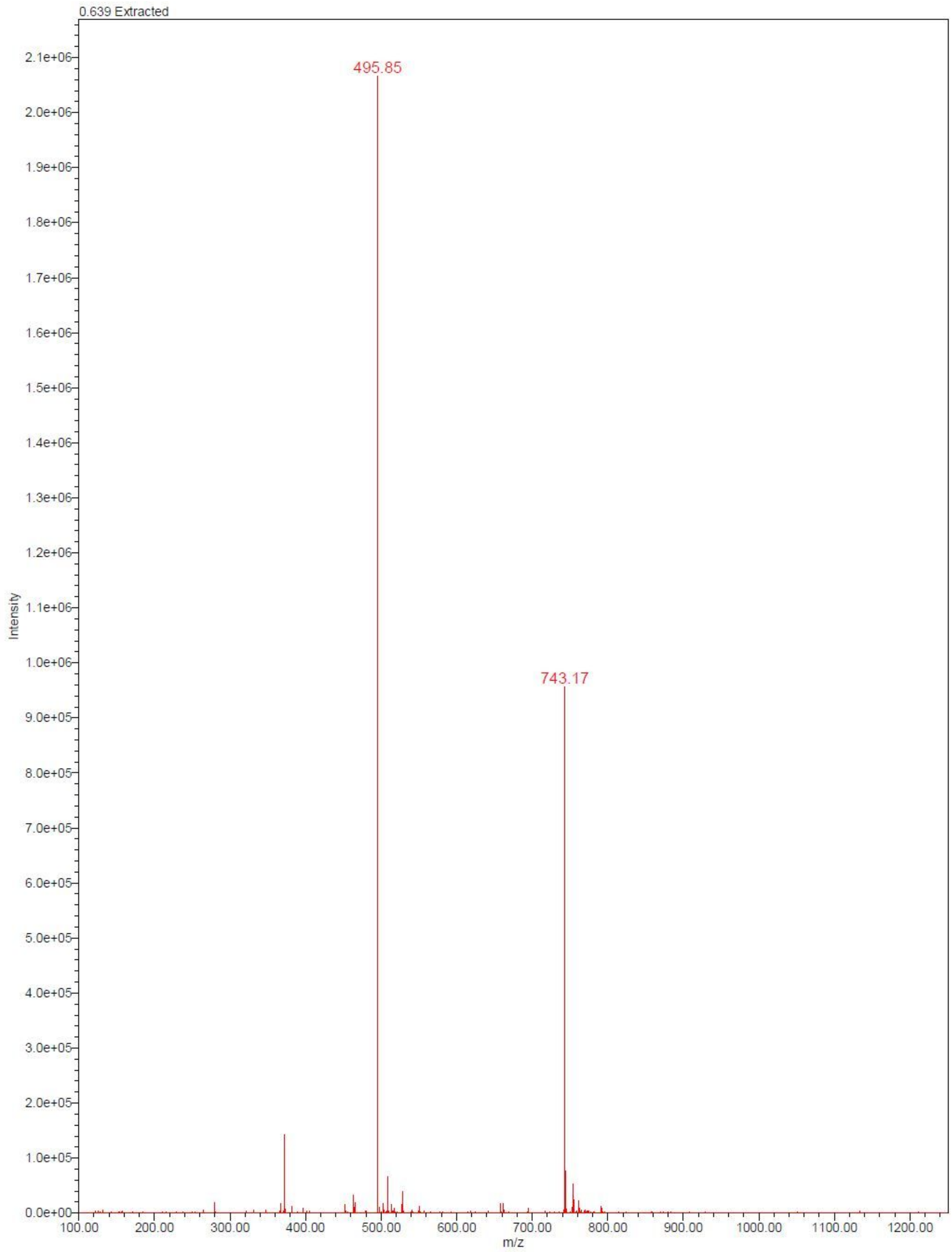

Figure S1. The MS spectrum of NT-20.3: calculated $[M+2H]^{2+}$ (*m/z*) 742.92; found 743.17.

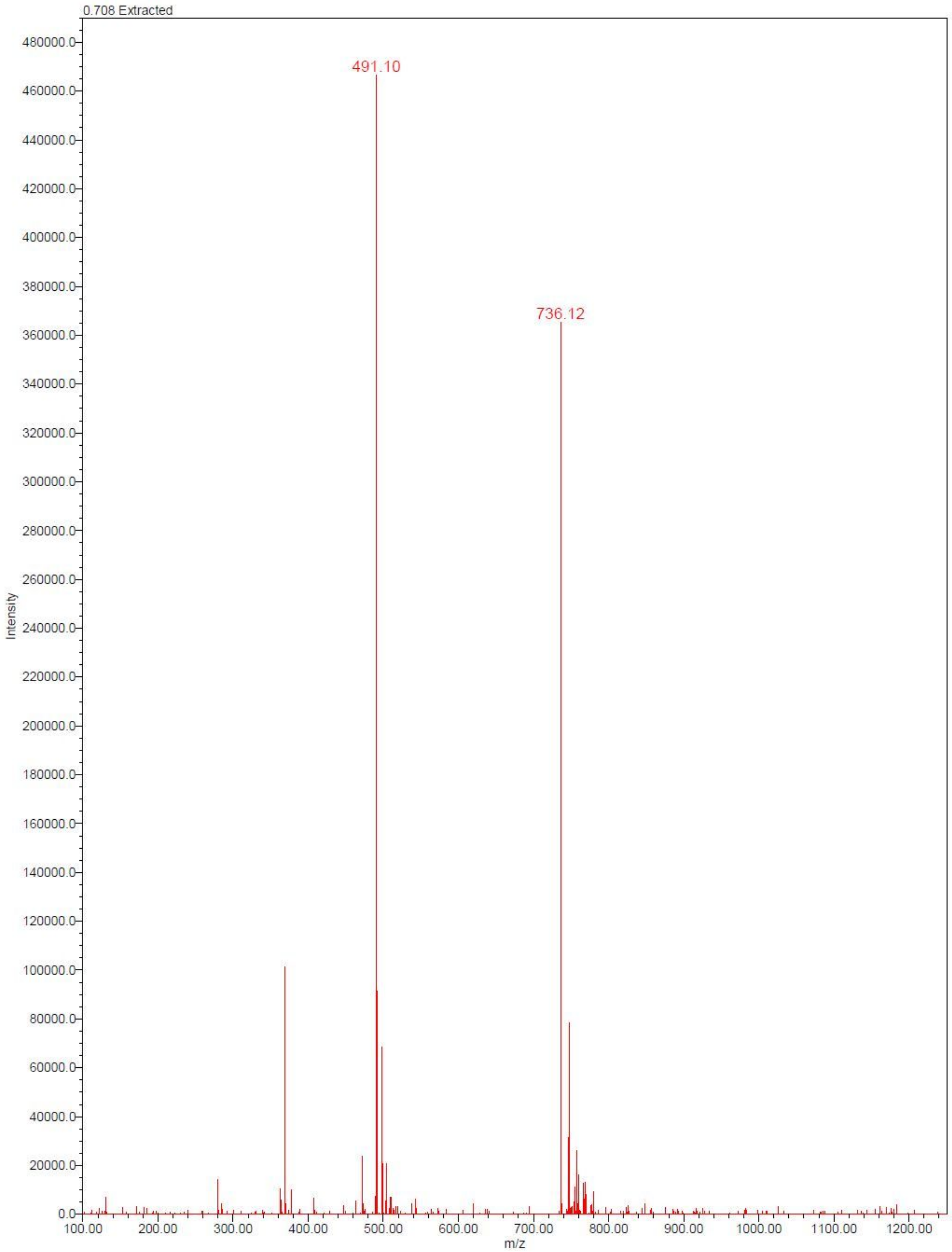

Figure S2. The MS spectrum of SK01001: calculated $[M+2H]^{2+}$ (*m/z*) 735.93; found 736.12.

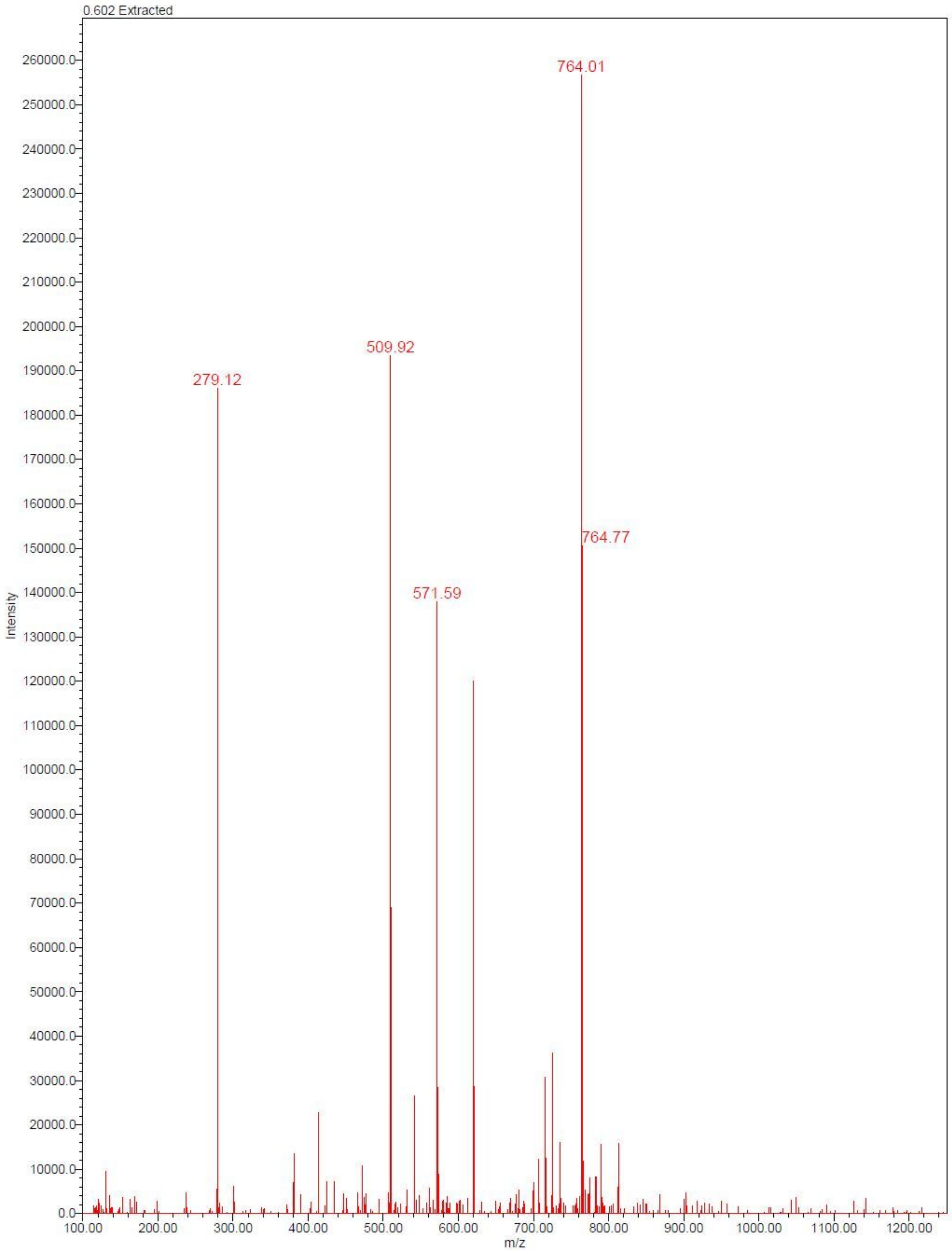

Figure S3.The MS spectrum of SK01003: calculated $[M+2H]^{2+}$ (*m/z*) 763.94; found 764.77.

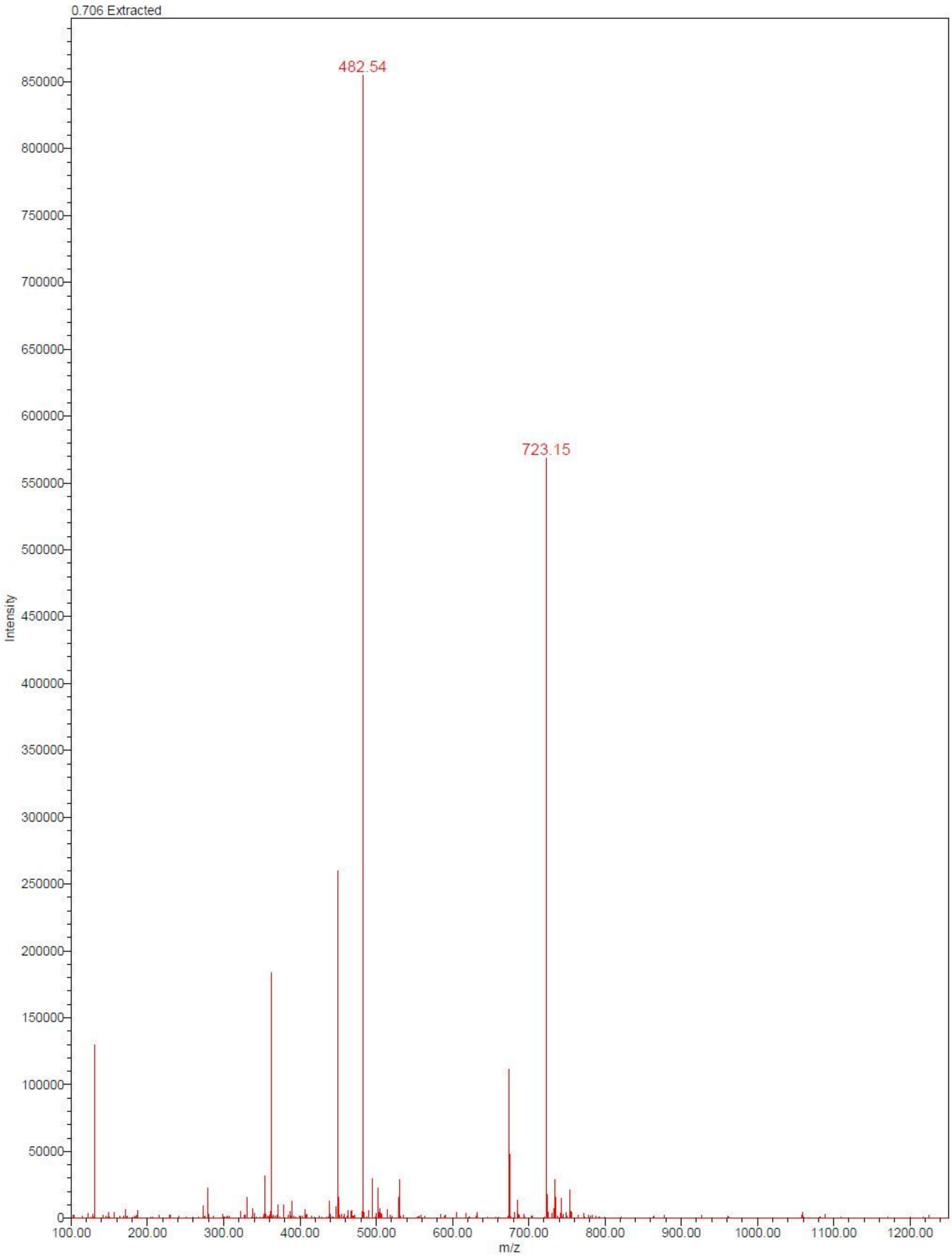

Figure S4. The MS spectrum of SK01005: calculated $[M+2H]^{2+}$ (*m/z*) 722.92; found 723.15.

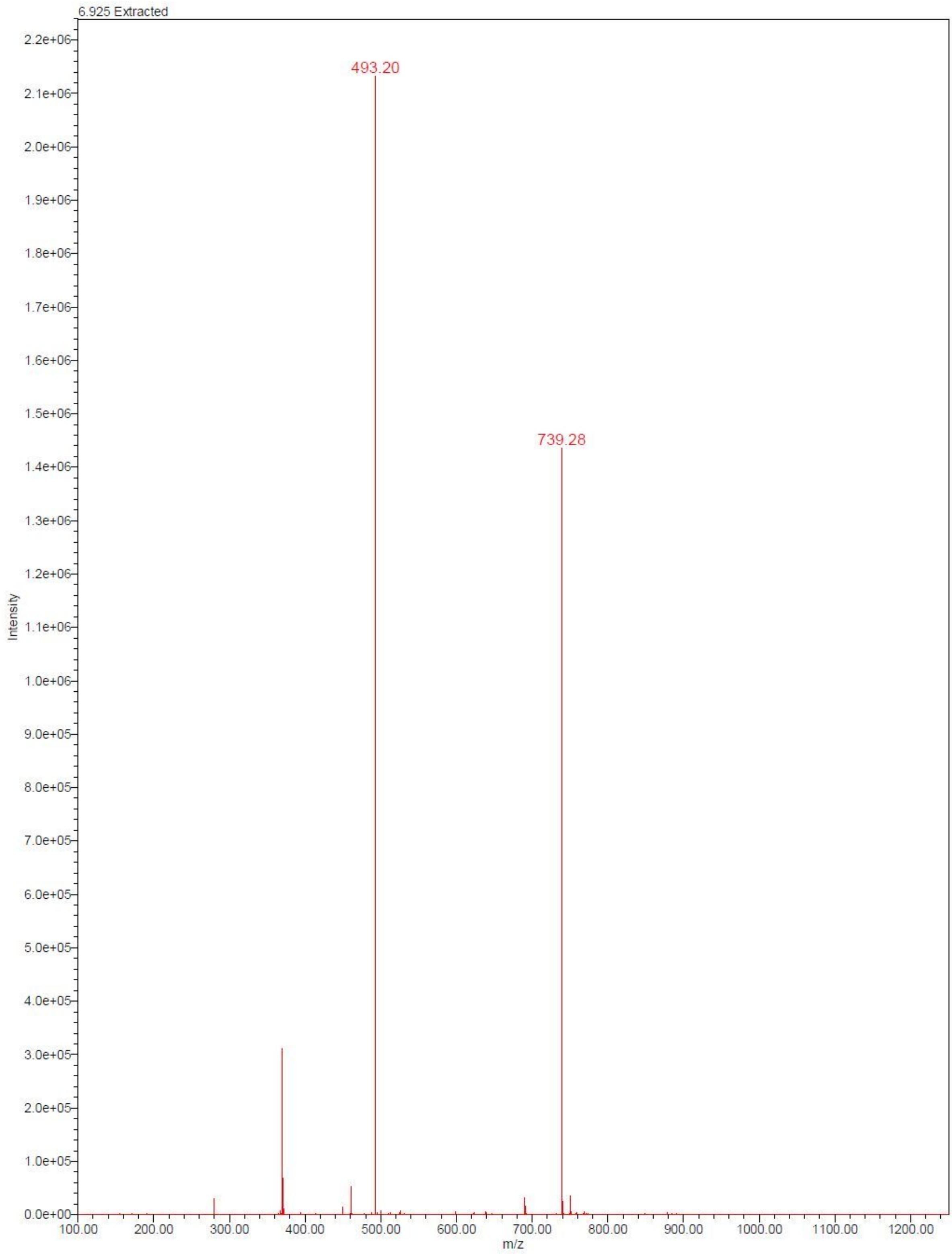

Figure S5. The MS spectrum of SM01010: calculated $[M+2H]^{2+}$ (*m/z*) 738.93; found 739.28.

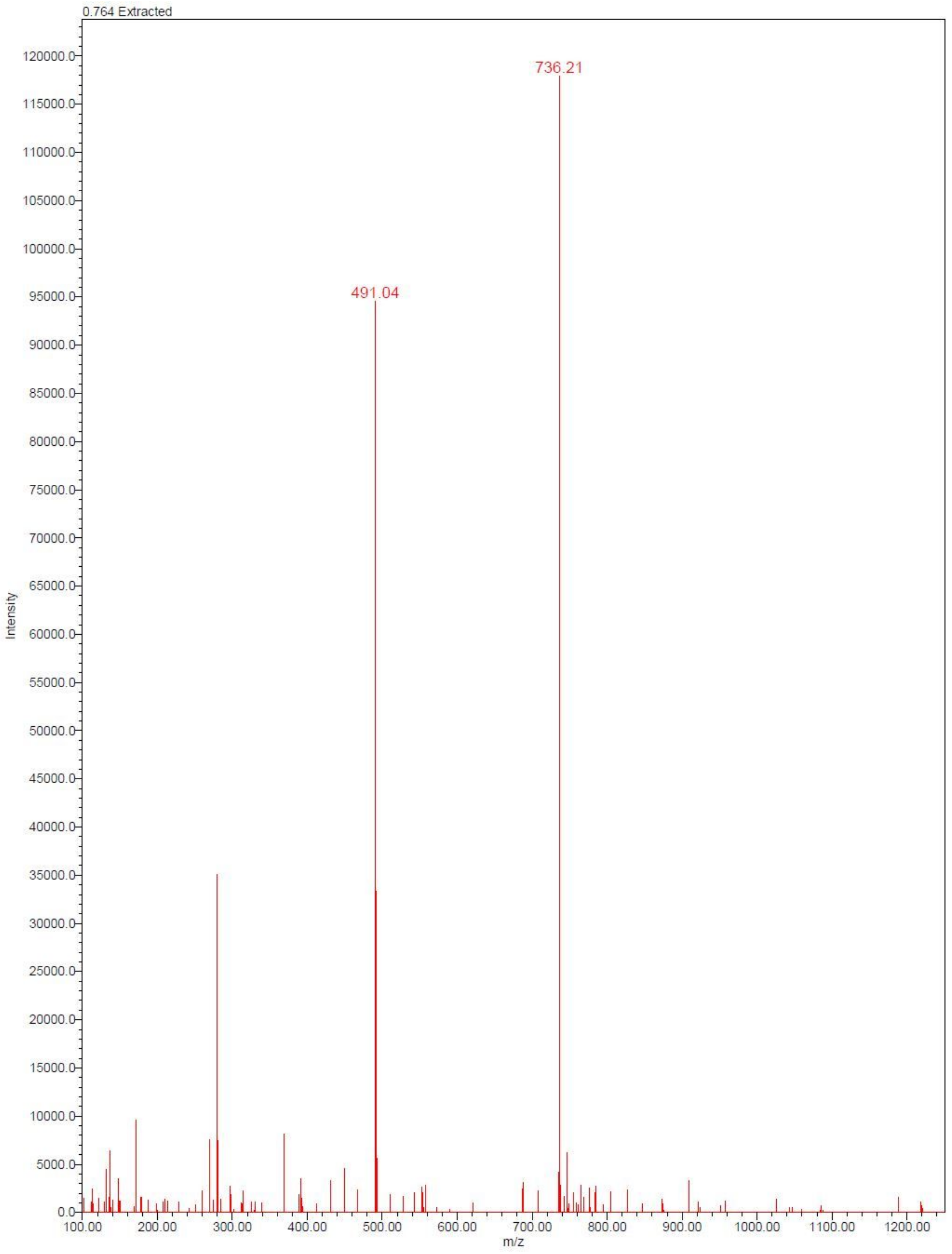

Figure S6. The MS spectrum of SK01012: calculated $[M+2H]^{2+}$ (*m/z*) 735.93; found 736.21.

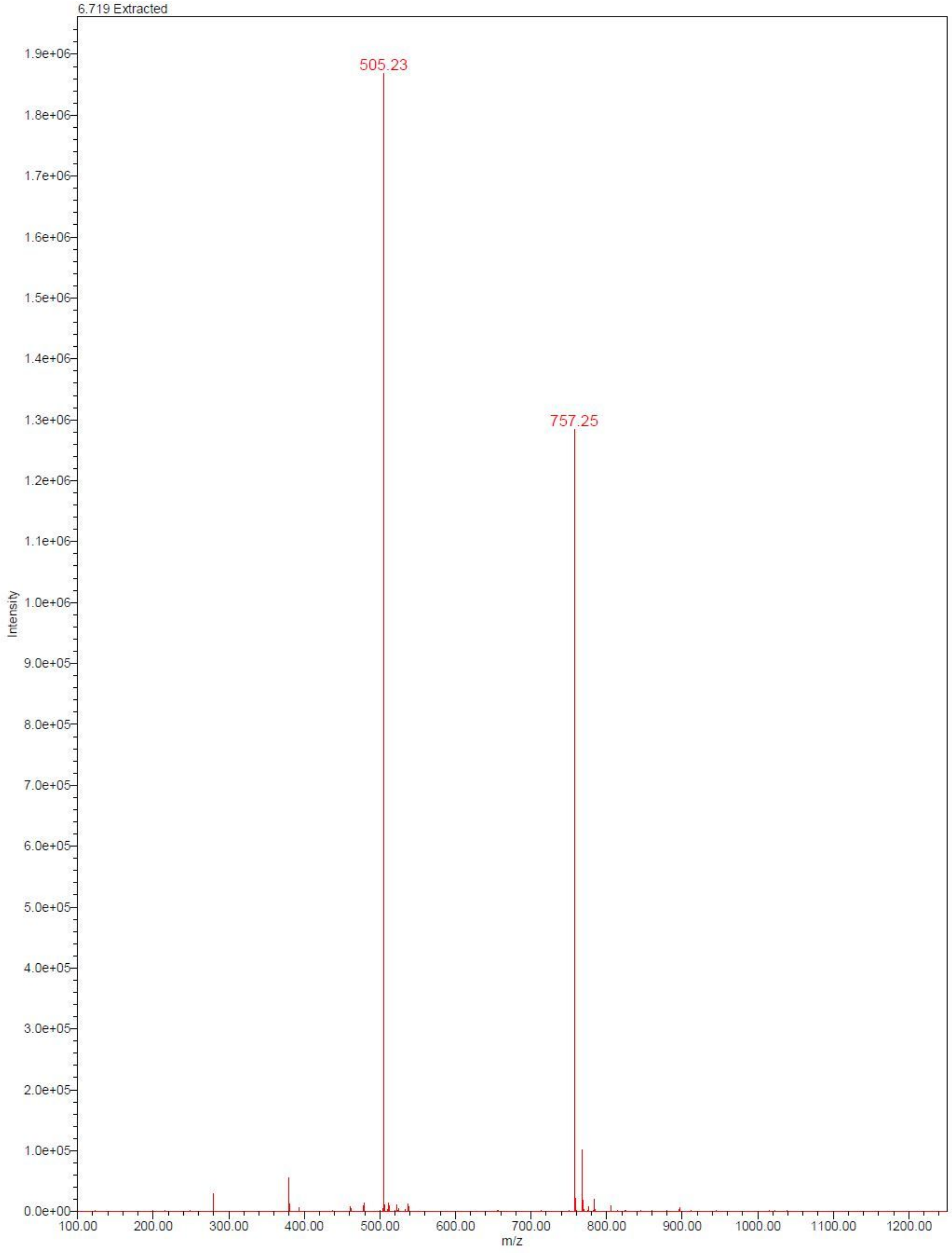

Figure S7. The MS spectrum of SK01014: calculated $[M+2H]^{2+}$ (*m/z*) 756.94; found 757.25.

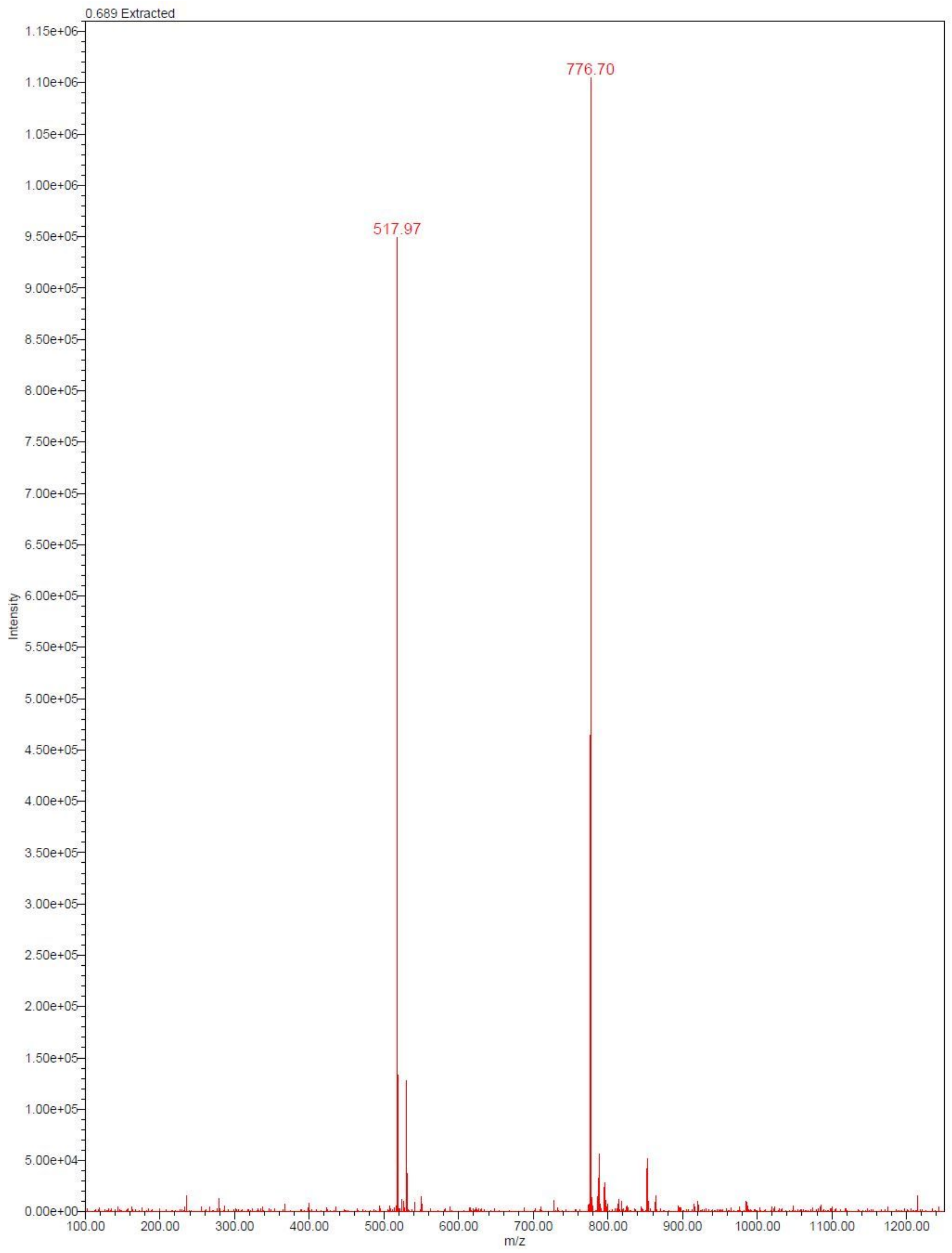

Figure S8. The MS spectrum of Ga-NT-20.3: calculated $[M+2H]^{2+}$ (*m/z*) 776.38; found 776.70.

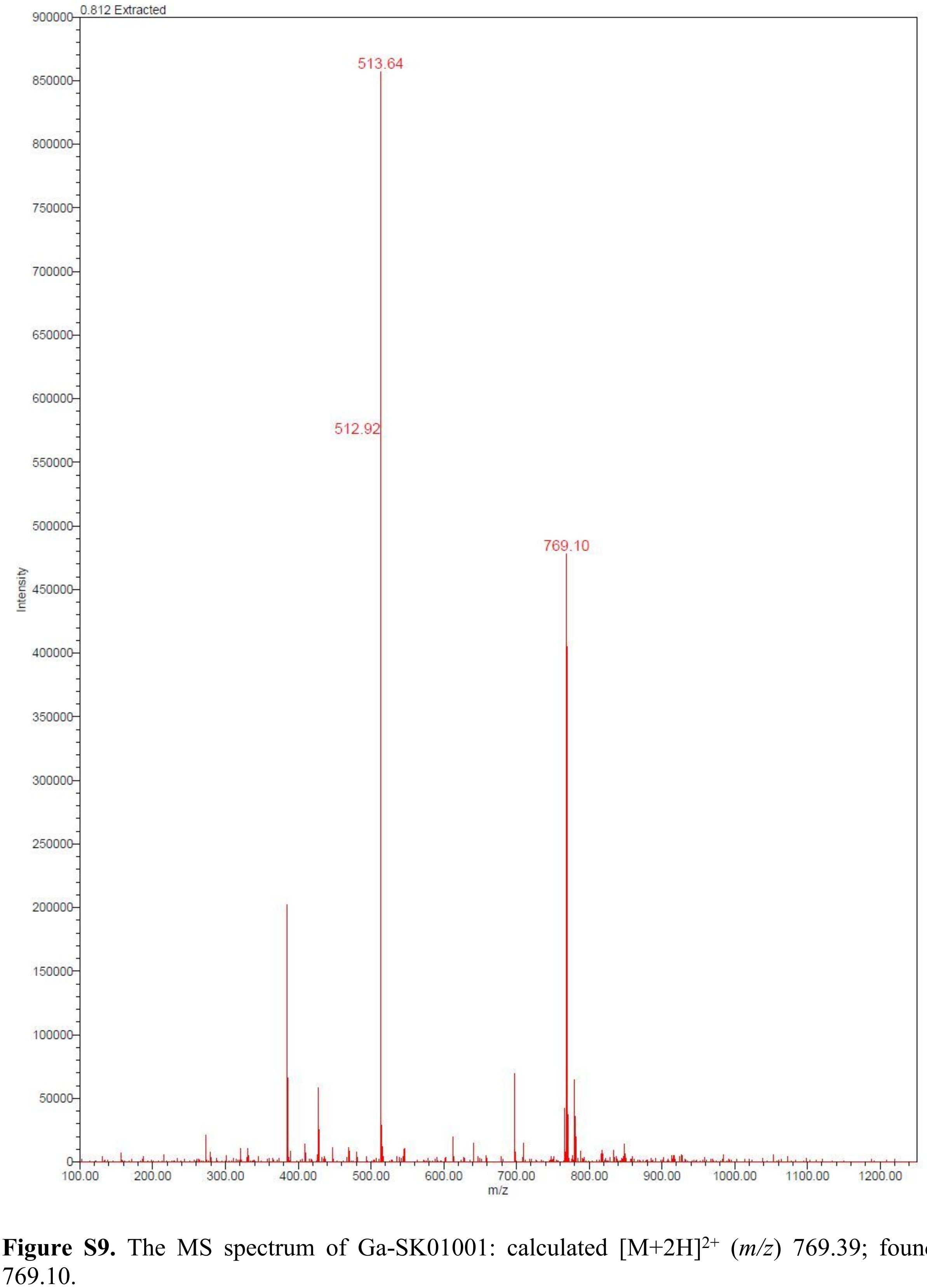

Figure S9. The MS spectrum of Ga-SK01001: calculated $[M+2H]^{2+}$ (*m/z*) 769.39; found 769.10.

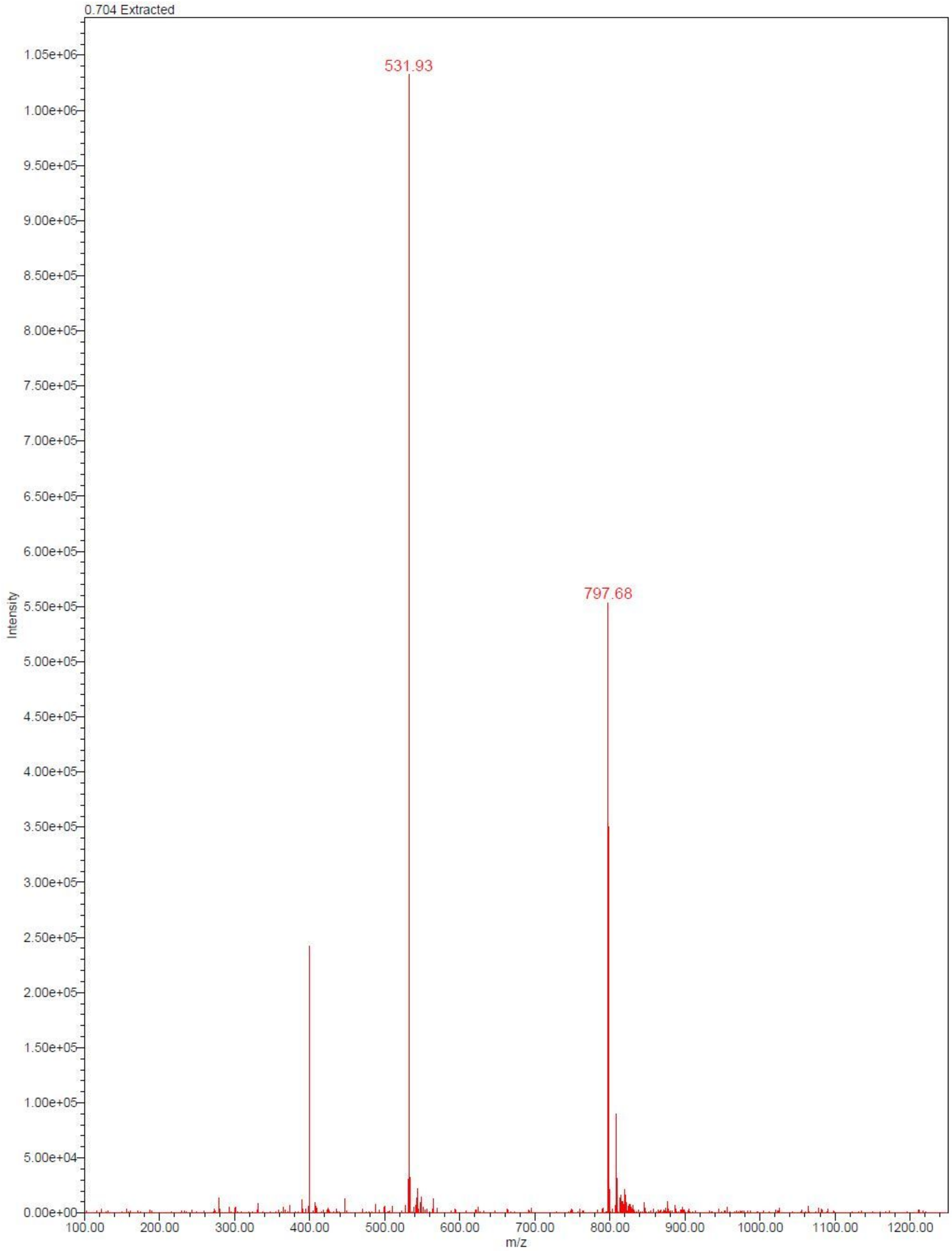

Figure S10. The MS spectrum of Ga-SK01003: calculated $[M+2H]^{2+}$ (*m/z*) 796.89; found 797.68.

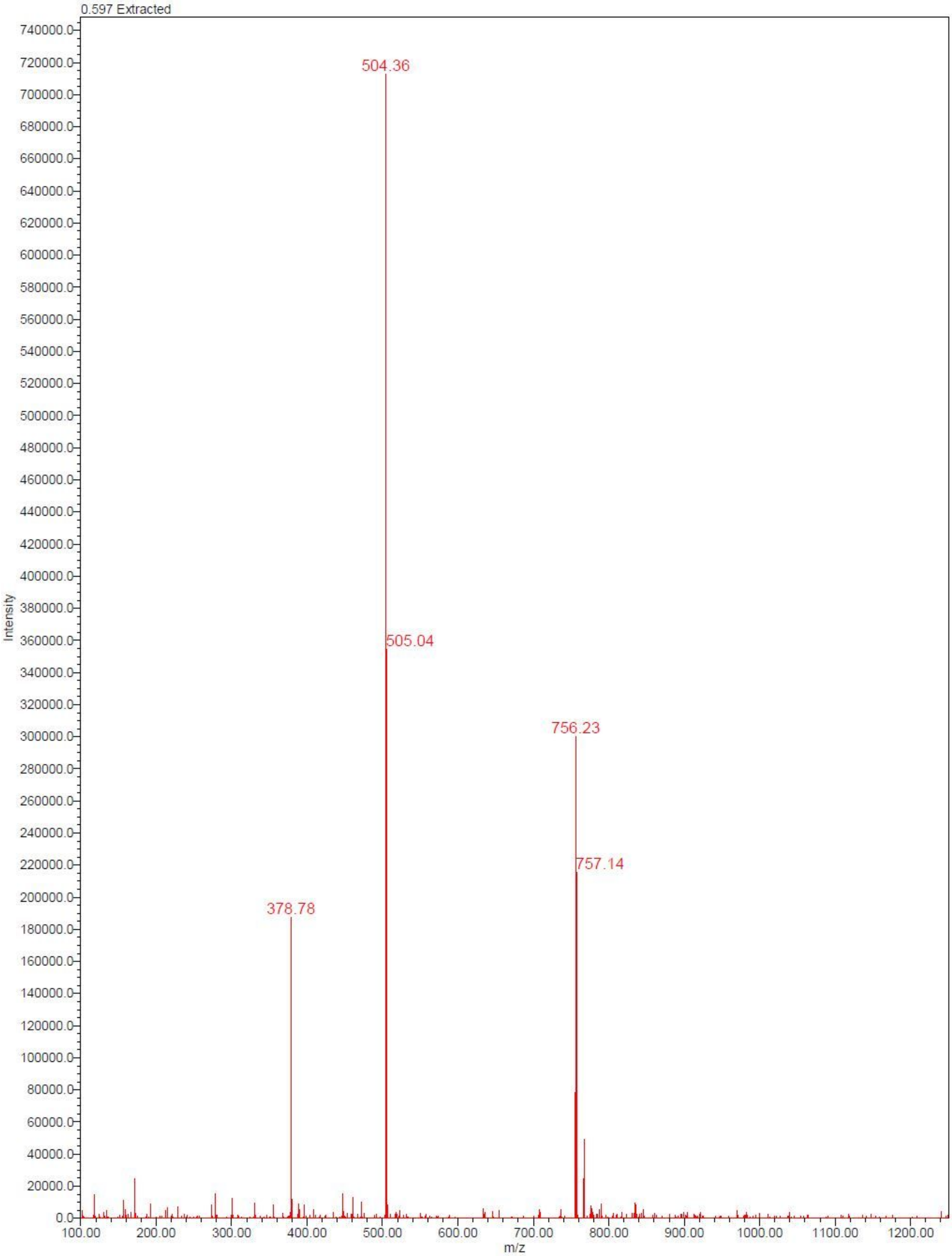

Figure S11. The MS spectrum of Ga-SK01005: calculated $[M+2H]^{2+}$ (*m/z*) 765.35; found 765.23.

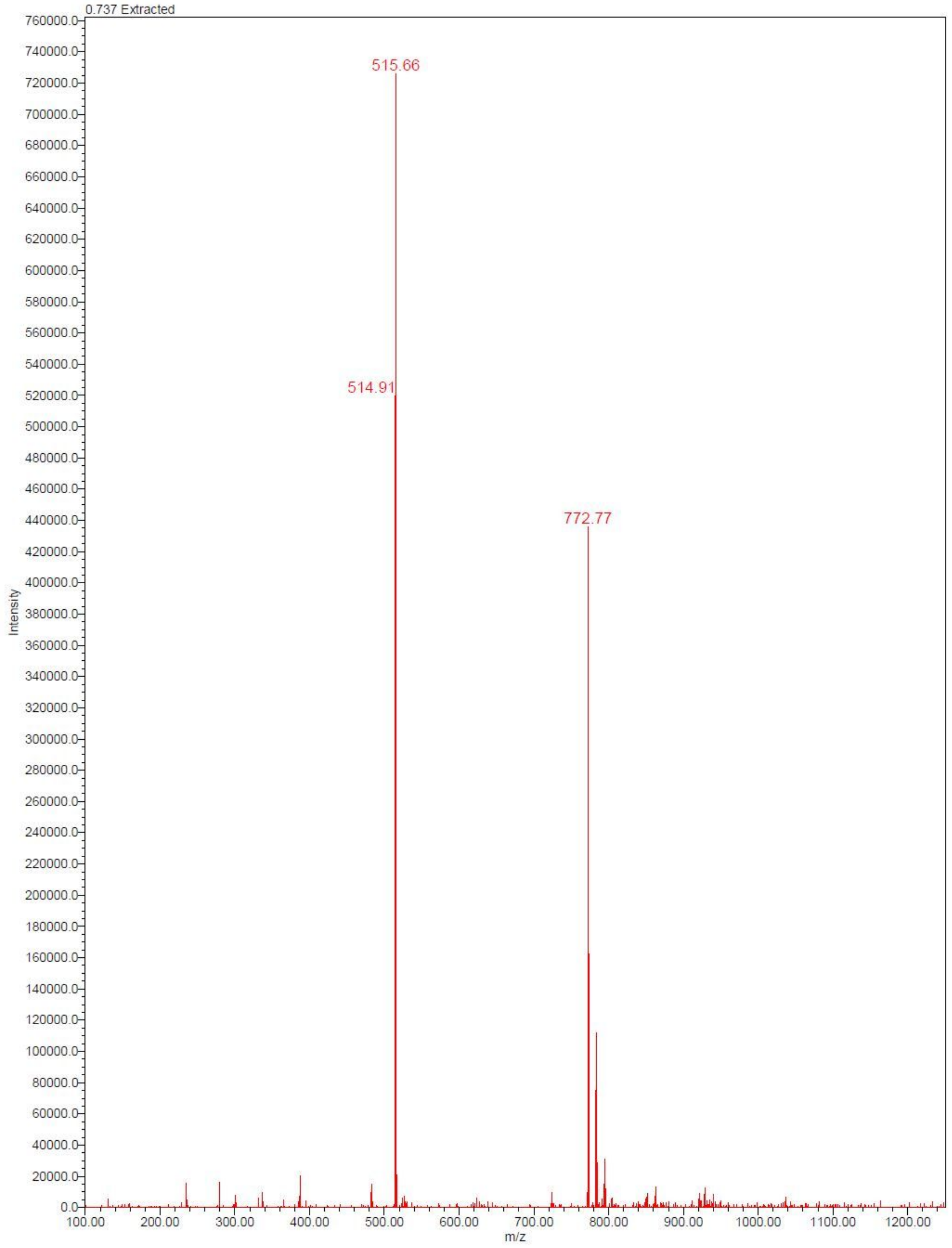

Figure S12.The MS spectrum of Ga-SM01010: calculated $[M+2H]^{2+}$ (*m/z*) 772.38; found 772.77.

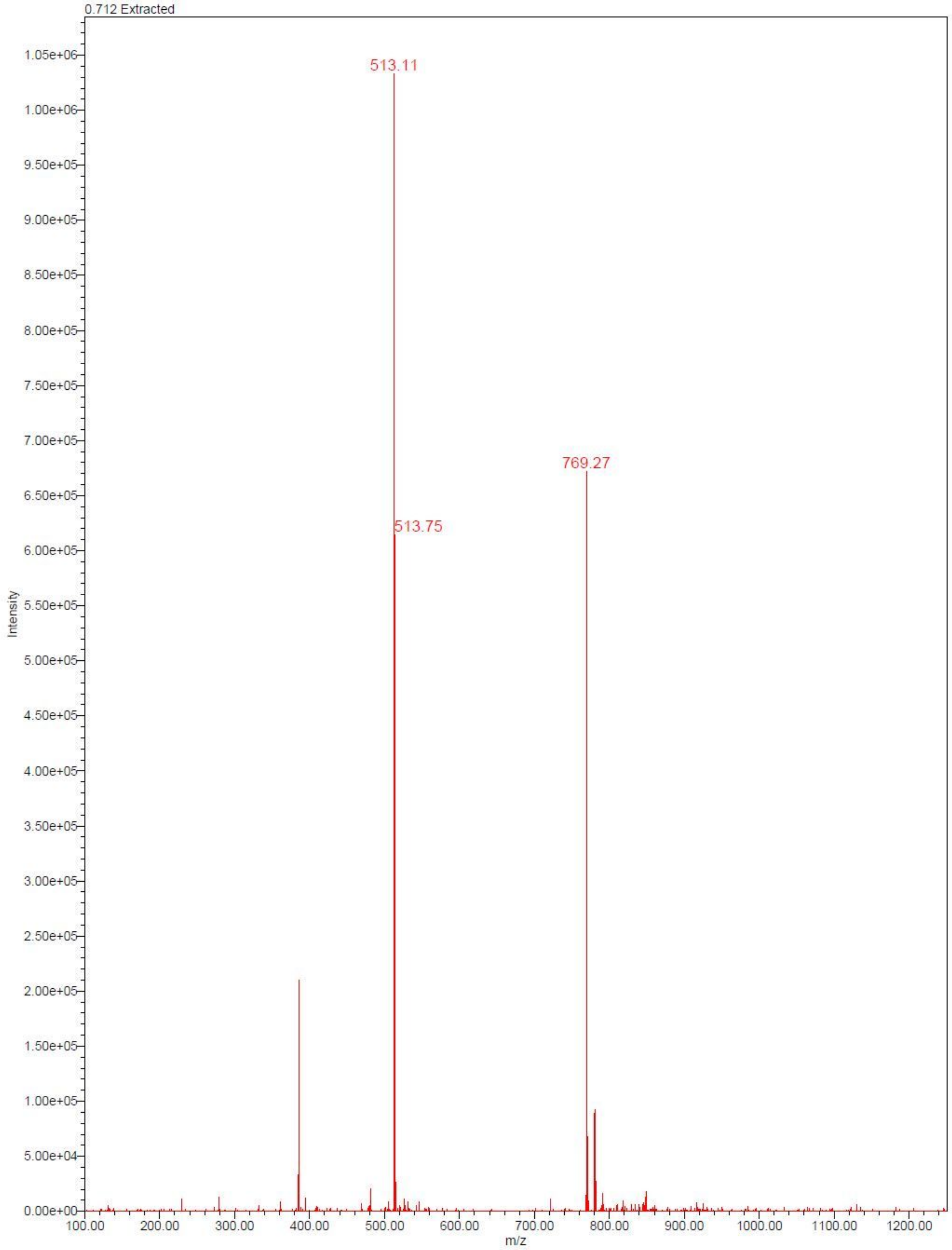

Figure S13. The MS spectrum of Ga-SK01012: calculated $[M+2H]^{2+}$ (m/z) 769.39; found 769.27.

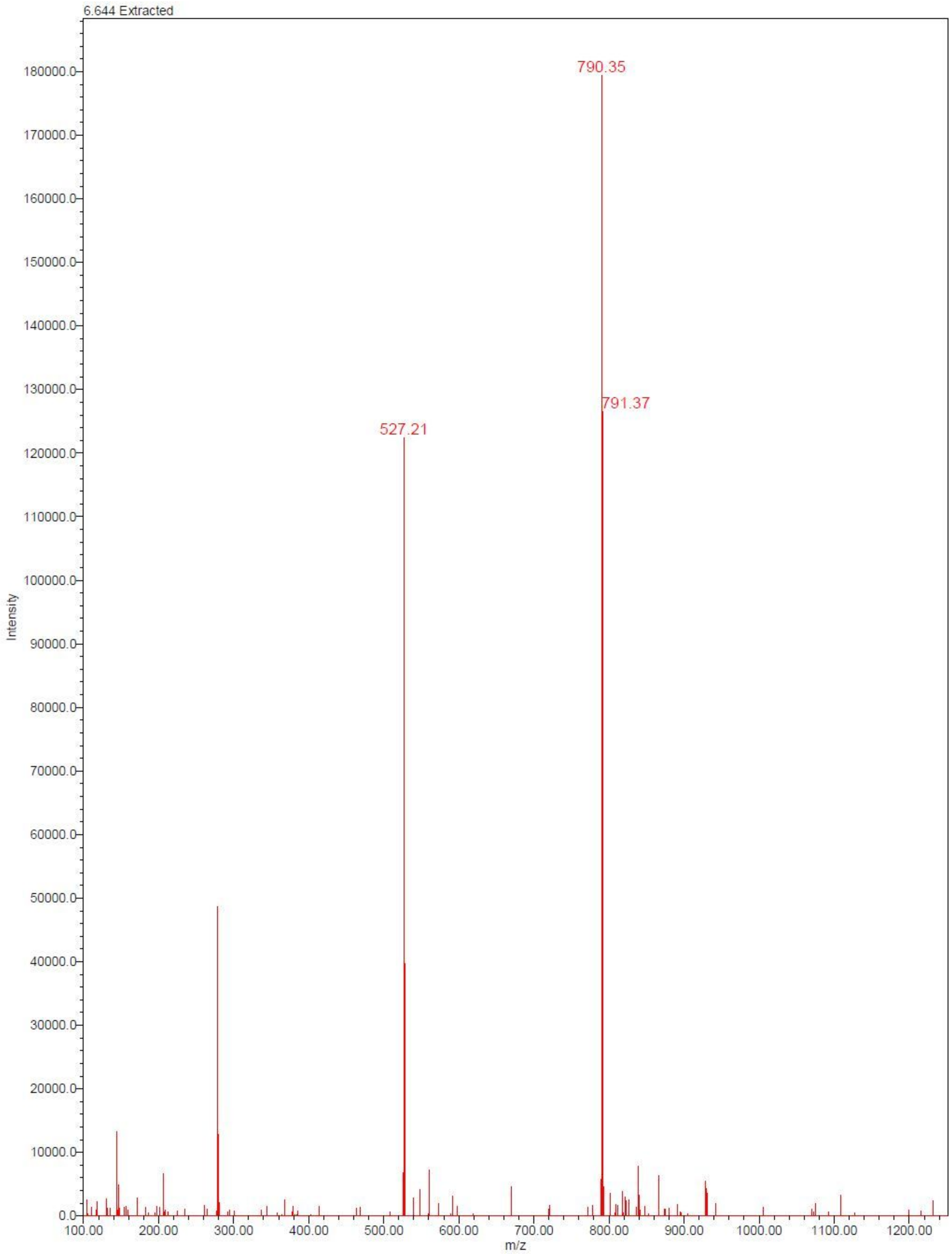

Figure S14. The MS spectrum of Ga-SK01014: calculated $[M+2H]^{2+}$ (*m/z*) 789.89; found 790.35.

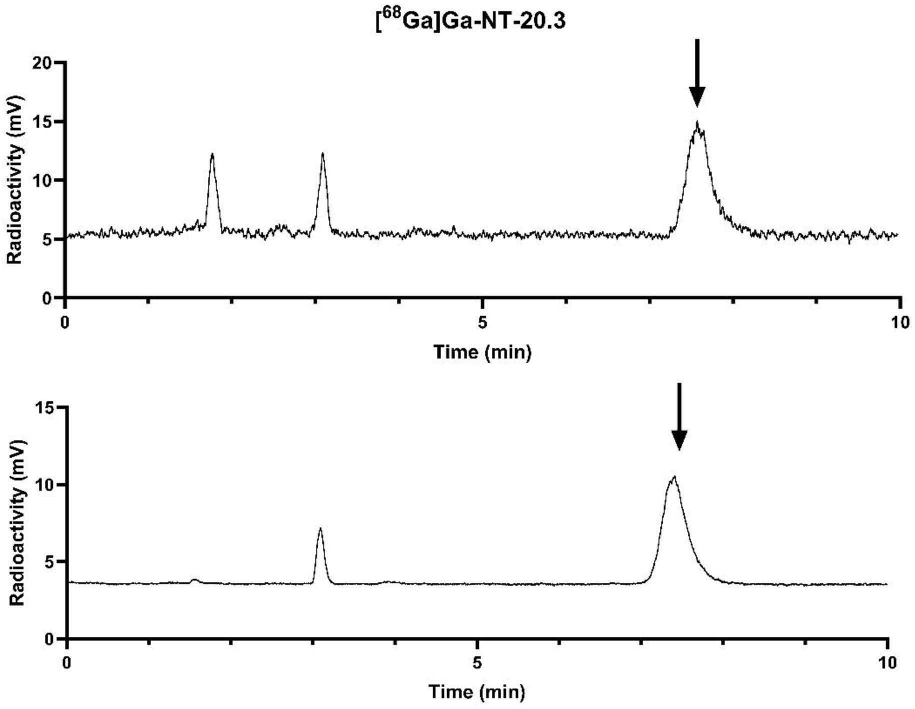

Figure S15. Radio-HPLC chromatograms illustrating the intact portions of [^{68}Ga]Ga-NT-20.3 detected in mouse plasma (top) and urine (bottom) at 15 minutes post-injection. The intact tracer peaks are marked with arrows.

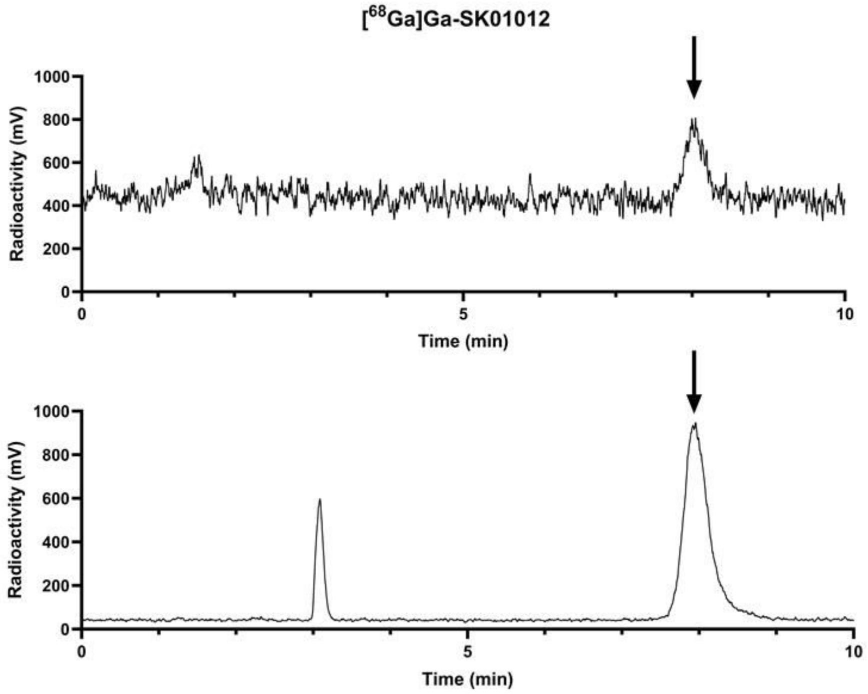

Figure S16. Radio-HPLC chromatograms illustrating the intact portions of [^{68}Ga]Ga-SK01012 detected in mouse plasma (top) and urine (bottom) at 15 minutes post-injection. The intact tracer peaks are marked with arrows.

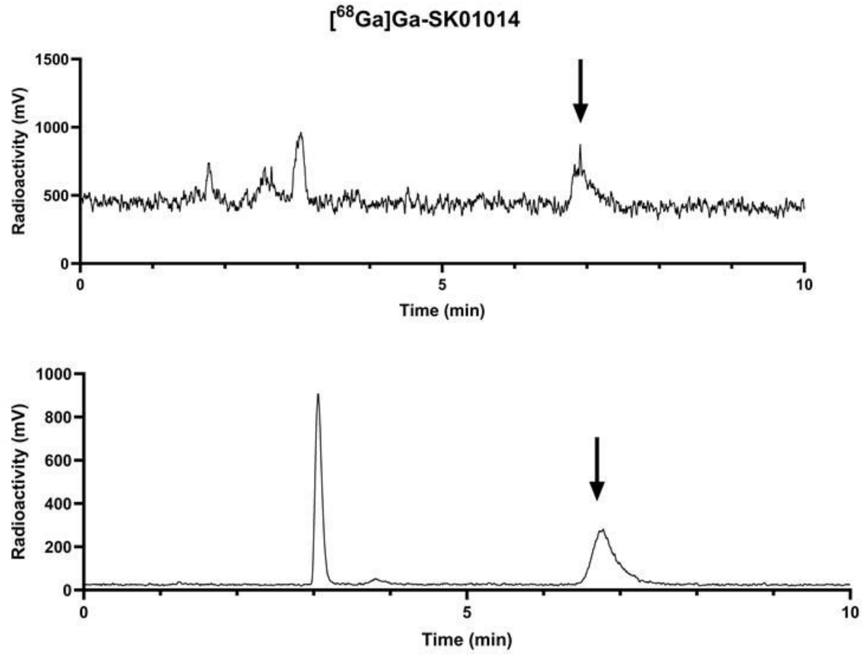

Figure S17. Radio-HPLC chromatograms illustrating the intact portions of [^{68}Ga]Ga-SK01014 detected in mouse plasma (top) and urine (bottom) at 15 minutes post-injection. The intact tracer peaks are marked with arrows.
